# Targeting of Alpha-V Integrins Reduces Malignancy of Bladder Carcinoma

**DOI:** 10.1371/journal.pone.0108464

**Published:** 2014-09-23

**Authors:** Geertje van der Horst, Lieke Bos, Maaike van der Mark, Henry Cheung, Bertrand Heckmann, Philippe Clément-Lacroix, Giocondo Lorenzon, Rob C. M. Pelger, Rob F. M. Bevers, Gabri van der Pluijm

**Affiliations:** 1 Department of Urology, Leiden University Medical Centre, Leiden, The Netherlands; 2 Galapagos SASU, Romainville, France; Centro Nacional de Investigaciones Oncológicas (CNIO), Spain

## Abstract

Low survival rates of metastatic cancers emphasize the need for a drug that can prevent and/or treat metastatic cancer. αv integrins are involved in essential processes for tumor growth and metastasis and targeting of αv integrins has been shown to decrease angiogenesis, tumor growth and metastasis. In this study, the role of αv integrin and its potential as a drug target in bladder cancer was investigated. Treatment with an αv integrin antagonist as well as knockdown of αv integrin in the bladder carcinoma cell lines, resulted in reduced malignancy *in* ****** *vitro*, as illustrated by decreased proliferative, migratory and clonogenic capacity. The CDH1/CDH2 ratio increased, indicating a shift towards a more epithelial phenotype. This shift appeared to be associated with downregulation of EMT-inducing transcription factors including SNAI2. The expression levels of the self-renewal genes NANOG and BMI1 decreased as well as the number of cells with high Aldehyde Dehydrogenase activity. In addition, self-renewal ability decreased as measured with the urosphere assay. In line with these observations, knockdown or treatment of αv integrins resulted in decreased metastatic growth in preclinical *in* ****** *vivo* models as assessed by bioluminescence imaging. In conclusion, we show that αv integrins are involved in migration, EMT and maintenance of Aldehyde Dehydrogenase activity in bladder cancer cells. Targeting of αv integrins might be a promising approach for treatment and/or prevention of metastatic bladder cancer.

## Introduction

Metastasis is a multistep process including invasion of the surrounding tissue, intravasation, survival in the bloodstream, extravasation and colonization of distant sites [1]. For the first steps in this process, cancer cells frequently switch from a sessile, epithelial phenotype towards a motile, mesenchymal phenotype, a process called epithelial-to-mesenchymal transition (EMT). In cancer, aberrant activation of this latent embryonic program contributes to progression to metastatic disease and therapeutic resistance, enabling cancer cells to become invasive, disseminate, resist apoptosis, stimulate angiogenesis and acquire stem/progenitor cell properties [2]–[5]. For the later stages of metastasis formation (e.g. colonization of a distant site), however, the reverse process of mesenchymal-epithelial transition (MET) may be required [2]–[5]. The critical involvement of epithelial plasticity (i.e. EMT and MET) along the metastatic cascade are best illustrated by differences in metastatic potential of phenotypical epithelial or mesenchymal bladder cancer cells in preclinical models *in vivo*. At the orthotopic inoculation site, representative for growth at the primary site and the multistep metastatic process, a mesenchymal phenotype is favorable for metastasis formation. However, after systemic (intracardiac) or intrabone inoculation, thus circumventing the first steps of metastasis formation, a more epithelial phenotype seems favorable [6].

Accumulating experimental and clinical evidence suggests that EMT can generate cells with stem/progenitor-like properties and enables plasticity between cancer stem cells (CSC) and non-CSC [4], [7]–[9], thus providing a link between EMT and CSCs [10], [11].

Tumor progression is driven by a subpopulation of cancer cells, the CSCs or tumor-initiating cells that have the ability to self-renew and to regenerate the phenotypic heterogeneity of the original tumor. Furthermore, CSCs have been shown to be involved in drug resistance, colonization and metastasis of distant organs [12]–[14]. To date, only a limited number of potential CSC markers have been described [15]–[21]. Recently, high aldehyde dehydrogenase activity (ALDH^hi^) was added to that list. The ALDH^hi^ subpopulation of cancer cells was found to be enriched in CSCs and to be involved in metastasis formation in several solid cancers, including breast [22], ovarian [23] and prostate [24] cancer. In bladder cancer, ALDH^hi^ cells showed a 100-fold increased heterogeneous tumor formation after subcutaneous inoculation in immuno-compromised mice as compared to ALDH^l^°^w^ cells [25].

During the process of carcinogenesis, which is often enabled by EMT, disseminated cancer cells seem to acquire self-renewal capability, similar to that displayed by stem cells [10] [11]. This raises the possibility that the EMT process may also impart a self-renewal capability to disseminated cancer cells. During EMT, epithelial markers - including CDH1 (E-cadherin) - are shed while mesenchymal markers, like VIM (vimentin), CDH2 (N-cadherin) and ITGAV/B5 (α_v_β_5_ integrin) are upregulated [3]. ITGAV receptors have been shown to be upregulated in cancer in both carcinoma cells and activated endothelial cells [26].

As shown previously by our group, the EMT process in prostate cancer cells could be reversed by adding an integrin receptor antagonist called GLPG0187 [27]. This non-peptide RGD antagonist blocks 6 integrin receptors; all five known αv integrin receptors with high affinity and ITGA5/B1 (α5β1) with lower affinity. Targeting of integrins by GLPG0187 inhibited the *de novo* formation and progression of bone metastases in prostate cancer by antitumor (including inhibition of EMT and the size of the prostate cancer stem cell population), antiresorptive, and antiangiogenic mechanisms [27]. GLPG0187 has also been shown to inhibit formation and progression of metastasis in breast cancer [28]. Integrin receptor antagonists, in the form of RGD-antagonists or antibodies, have been shown to decrease angiogenesis, tumor growth and metastasis in several solid tumor types in which ITGAV is upregulated, including breast cancer, melanoma and prostate cancer. [27], [29], [30]. Several of these antagonists are currently in phase I and II clinical trials [26], [31]. GLPG0187 is currently in phase Ib clinical trial for patients with a variety of solid tumors. The effect of blocking integrin receptors by GLPG0187 were similar to effects of knockdown of ITGAV in prostate cancer cells. These data indicate that ITGAV is functionally involved in the migratory, mesenchymal cellular phenotype of prostate cancer cells. Moreover, ITGAV is important for the acquisition of prostate cancer cells with a metastasis-initiating capacity [32].

Inhibition of α_v_ integrin might also have therapeutic potential in bladder cancer, since ITGAV is significantly overexpressed in bladder tumors (46%) compared to normal urothelium (13%) and a trend is observed of stage and grade-dependent increase in ITGAV expression [33].

In the present study we determined the effect of functional inactivation of ITGAV (targeting with GLPG0187 or knockdown of ITGAV) on migration, EMT and stemness in bladder cancer using the human bladder carcinoma cell line UM-UC-3 and the human papilloma cell line RT-4.

Functional inactivation of ITGAV in bladder cancer leads to a less malignant phenotype as illustrated by significantly impaired migration, EMT response, clonogenicity and a reduction in the size of the stem/progenitor pool. In line with these *in vitro* observations, knockdown of ITGAV or treatment with GLPG0187 significantly inhibited metastasis and secondary tumor growth (in bone marrow).

These data indicate that ITGAV inhibition represents a novel, promising strategy for the prevention and/or treatment of bladder cancer growth and metastasis.

## Materials and Methods

### Cell lines and culture conditions

The bladder carcinoma cell line UM-UC-3 and the bladder papilloma cell line RT-4 were obtained from ATCC (catalog no.CRL-1749 and HTB-2). UM-UC-3 cells were routinely cultured in ATCC Eagle’s Minimal Essential Medium (ATCC) and RT-4 cells in McCoy’s 5A+Glutamax medium (Invitrogen Life Sciences, Bleiswijk, the Netherlands), both supplemented with 10% fetal bovine serum (FBS), 100 units/ml penicillin (Invitrogen) and 50 µg/ml streptomycin (Invitrogen). The UM-UC-3 cell line was stably transfected with pCAGGS3.1 luciferase 2 (modified pGI4 luciferase 2 vector (Promega, The Netherlands)) as previously described [34], resulting in the UM-UC-3luc2 cell line, which was maintained in medium supplemented with 0.8 mg/ml geneticin (Invitrogen). HEK293T cells were maintained in DMEM containing 10% FBS (Invitrogen). All cell lines were grown in a humidified incubator at 37°C and 5% CO_2_ and were regularly tested for mycoplasm.

### Suppressing ITGAV expression with a shRNA-lentiviral vector

UM-UC-3luc2 and RT-4 cell lines were transduced with short hairpin RNAi constructs against ITGAV or scrambled non-targeting (NT) shRNA derived from Sigma’s MISSION library (table S1). HEK293T cells were transfected with the short hairpin constructs together with the packaging plasmids REV, GAG and VSV in a 1∶1∶1∶1 ratio using Fugene HD (Roche) as transfection reagent. Cells were mixed with 1 ml lentiviruses containing the shRNA-lentiviral vector and 8 µg Polybrene (Sigma) was added. The mixture was incubated for 1–2 hours at RT. Cells stably expressing the shRNA were selected using puromycin (1 µg/ml, Sigma). The effects of ITGAV knockdown described in this manuscript, represent activities of the heterogeneous cell populations transduced with high efficiency by the lentivirus and not single-cell selected clones. The αv kd cell lines will further be referred to as sh clone 1 and 2 and the non-targeting control cell line as UM-UC-3luc2 NT cells and RT-4 NT cells.

### Migration assay

6×10^4^ pre-starved cells (1% FBS for 16 h) were seeded in the upper chamber of an 8-µm Transwell migration chambers (Costar, Corning incorporated, Corning, NY, USA) [24]. Cells were allowed to attach and then either vehicle or GLPG0187 was added. Cells were allowed to migrate for 6 h towards serum-containing medium in the lower chamber. Subsequently, cells were fixed with 4% paraformaldehyde (Merck, Darmstadt, Germany) and stained with 0.1% crystal violet (2 mg/ml, Sigma-Aldrich, The Netherlands). Four random fields were counted for each well and mean numbers of migrated cells/area of 4 fields were calculated (4 wells/condition, n = 3).

### Clonogenic assay

Cells were seeded in a 96-well plate at an average of 1 cell/well. After 14–16 days colonies were clearly visible and the size of the colonies and the mean numbers of colony-containing wells per plate were determined by light microscopy (Zeiss Televal 31, Germany) (3 plates/condition, n = 3).

### Urosphere assay

UM-UC-3 cells were seeded 100 cells/cm^2^ in an ultra-low attachment plate (Corning) in serum-starved conditions (DMEM F12 supplemented with N2 and B27) [35]. The percentage of cells with sphere forming capacity (P0) was measured after 10 days of culture. P0 spheres were dissociated into single cells and seeded in ultra-low attachment 96 wells. The percentage of cells with sphere forming capacity (subsequently P1–3) was measured 10 days after seeding spheres dissociated into single cells. The area of the spheres was measured with Image J software.

### Flow cytometry

Cells surface stainings were performed by labeling cells for 45 min at 4°C in the dark (1∶10 in PBS containing 0.1% sodium azide and 1% FBS) (table S2). Intracellular stainings were performed by fixing cells in ice-cold methanol, followed by washing with PBS and incubation with 0.5% saponin. Cells were labelled for 30 min at RT, followed by washing and secondary antibody (goat anti-rabbit Alexafluor 488) for 30 min at RT in the dark.

Relative expression levels are measured as % of positive cells * mean fluorescence intensity of each condition compared to either the NT or vehicle treated cells. ALDH activity was measured using the ALDEFLUOR assay kit (Stem cell technologies, Aldagen Inc., Durham, NC, USA) as described before [24] (n = 3).

### RNA isolation and real-time qPCR

RNA was extracted using Tripure isolation reagent (Roche Diagnostics GmbH, Manheim, Germany) according to manufacturer’s instructions. Real-time qPCR was run and analyzed with a Biorad IQ5 cycler (Biorad, Veenendaal, The Netherlands). For primer sequences see table S3. Gene expression was measured relative to GAPDH expression using the following formula: log2^−ΔΔCt^.

### Xenograft experiments

#### Mouse Strain

Female nude mice (Balb/c nu/nu; Charles River, L’Arbresle, France) were housed in individual ventilated cages under sterile condition according to the Dutch guidelines for the care and use of laboratory animals. The protocol was approved by the Committee on the Ethics of Animal Experiments of the Leiden University, The Netherlands (DEC 11082).

#### Intracardiac Inoculation

A single cell suspension of 1×10^5^ UM-UC-3luc2 cells/100 µl PBS was injected into the left cardiac ventricle of 4-week old nude mice as described previously [34].

#### Intraosseous inoculation

Two holes, 4 to 5 mm apart, were drilled through the bone cortex of the upper tibia with the aid of a 25-gauge needle (25G ^5^/_8_, BD Micro-Fine, Becton Dickinson, Etten-Leur, The Netherlands). Space in the bone marrow was created by flushing out the bone marrow from the proximal end of the shaft using a 30-gauge needle (30G ½, BD Micro-Fine, Becton Dickinson). Subsequently, a single cell suspension of 1×10^5^ UM-UC-3luc2 cells/10 µl PBS was injected into the tibia of 4-week old nude mice via a 30-gauge needle (30G ½, BD Micro-Fine). Finally, the cutaneous wound was sutured.

#### Bioluminescence imaging

Bioluminescence imaging (BLI)was performed using the IVIS Lumina Imaging System (Caliper LifeSciences, USA) [34]. Images were quantified with Living Image and values expressed as relative light units (RLU). Tumor take was measured as the % of mice with BLI foci.

### Statistical analysis

Data are presented as mean ± SEM. Two-way ANOVA’s were performed followed by the post-hoc Bonferroni test (SPSS20). A P-value <0.05 was considered significant (*P<0.05, **P<0.01, ***P<0.001).

## Results

### Characteristics of αv integrin knockdown and GLPG0187 treated bladder cancer cell lines

**Figure 1:**
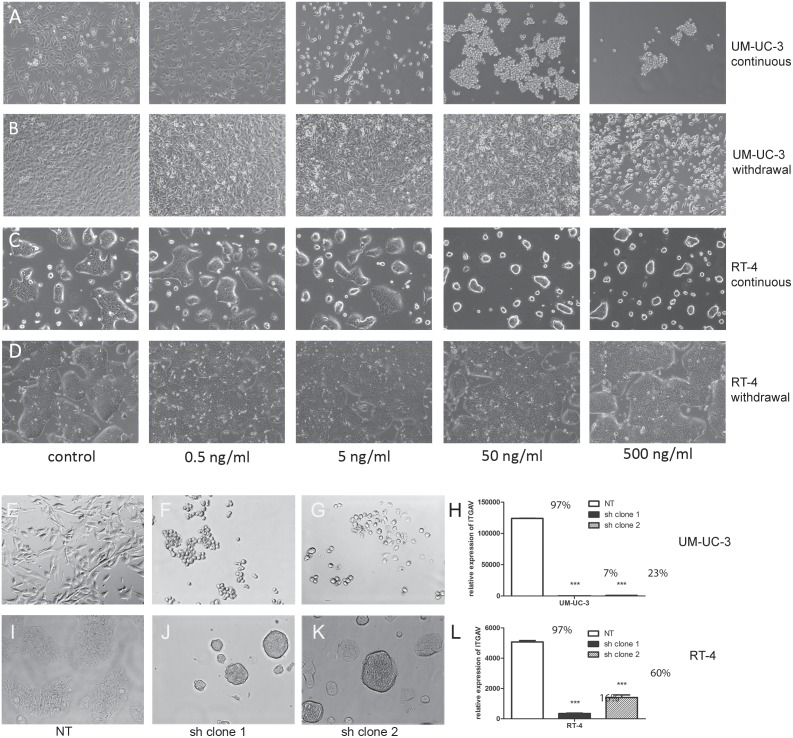
Effects of GLPG0187 and ITGAV knockdown on adherence to tissue culture plastic. Representative images of cells treated for 24 hours with a concentration series of GLPG0187 (dosage between 0–500 ng/ml, indicated underneath the images). Treatment resulted in a dose-dependent loss of adherence to tissue culture plastic in both UM-UC-3luc2 cells (A) and RT-4 cells (C). After 48 hours of GLPG0187 treatment, cells cultured for 4 days in GLPG0187-free medium regained their adherence to the tissue culture plastic in UM-UC-3luc2 cells (B) and RT-4 cells (D). Loss of adherence was also observed in UM-UC-3luc2 and RT4 cells stably transduced with a short hairpin targeted against ITGAV (respectively F–G for UMUC3luc2 sh ITGAV clones 1 and 2 and J–K for RT4 shITGAV clones 1 and 2). As a control, cells stably transduced with a non-targeting short hairpin (NT) were used (UMUC3 (E) and RT4 (I)). Flow cytometric analysis of relative ITGAV expression levels in UM-UC-3luc2 (H) and RT4 (L) cells (% of positive cells * mean fluorescence intensity). Data are presented as mean ± SEM, n = 3, the percentage of ITGAV positive cells is indicated above the bars.

To determine the effect of α_v_ integrin on bladder cancer, two cell lines were used; the human carcinoma cell line UM-UC-3, representing invasive bladder tumors, and the human papilloma cell line RT-4, representing non-invasive bladder tumors. UM-UC-3 cells were stably transfected with *firefly* luciferase 2 (luc2), and can be used for sensitive *in vivo* cell tracking [34]. ITGAV (α_v_ integrin) is expressed both in normal urothelium and carcinoma (respectively Figure S1D and S1E). UM-UC-3luc2 and RT-4 cells were treated with the non-peptide RGD antagonist GLPG0187 in a concentration range of 0, 0.5, 5, 50 or 500 ng/ml. GLPG0187 treatment resulted in a dose-dependent detachment from the tissue culture plastic within 24 hours in both cell lines (respectively Figure 1A and C). No significant effect on viability was observed (Figure S1G) and the effects of GLPG0187 treatment proved to be largely reversible, as cells regained attachment to the tissue culture plastic when GLPG0187-free medium was provided after 48 h of GLPG0187 treatment (Figure 1B and D).

In parallel, ITGAV expression was blocked with 2 independent lentiviral-mediated shRNA constructs which resulted in 99% knockdown of ITGAV in UM-UC-3luc2 sh clone 1 and in sh clone 2, compared to the cells that were transduced with non-target (NT) shRNA (Figure 1H and Figure S1A and S1B). In RT-4 cell, knockdown was respectively 97% for sh clone 1 and 74% for sh clone 2 (Figure 1L and Figure S1A and S1C). Upon knockdown of ITGAV expression, both cell lines displayed strong morphological changes, closely resembling the effect of the non-peptide RGD antagonist GLPG0187. The cells were no longer adherent to tissue-culture plastic and grew in suspension where they clustered together and formed small clumps of cells (Figure 1 E-G UM-UC-3, I-K RT-4). This offered an additional means of selection, since only the cells with very low levels of ITGAV expression detached from the tissue culture plastic (Figure S1F). This effect was not due to reduced viability, since no significant difference in UM-UC-3luc2 viability was observed using Annexin V/PI apoptosis assays (Figure S1G).

Knockdown of ITGAV did not significantly affect the expression levels of ITGA2 in both UM-UC-3 and RT-4 cells (Figure S6A–C). A small decrease in ITGA6 protein expression was observed in RT-4 cells, whereas no effect on ITGA6 was measured in UM-UC-3 cells (Figure S6D–F).

### Effects of αv integrin on proliferation and migratory capacity

Subsequently, the effects of α_v_ kd and GLPG0187 treatment on characteristics that are required for tumor growth and metastasis formation such as proliferation, migration and clonogenic capacity were evaluated. Proliferative capacity was significantly decreased in the UM-UC-3luc2 sh clones (Figure S1H). A decrease, although not significant was also seen in the proliferative capacity of the RT-4 sh clones compared to NT cells (Figure S1I). GLPG0187 did show a significant decrease in proliferation rates of both cell lines 72 hours after treatment with the highest doses of GLPG0187 (i.e. 50 ng/ml and 500 ng/ml GLPG0187; Figure S1J and S1K).

**Figure 2:**
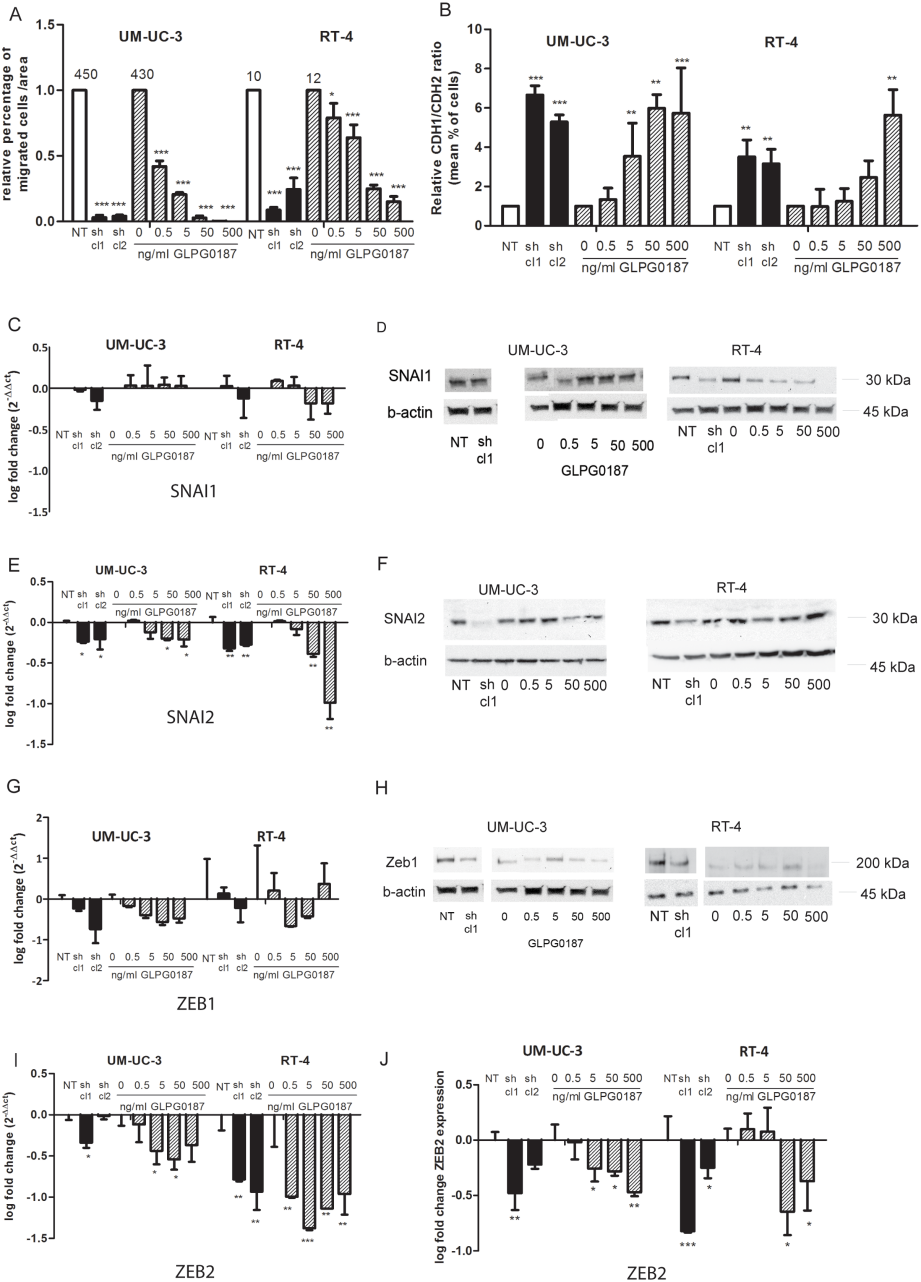
Effects of α_v_ integrin on migration and EMT. Effects of knockdown of ITGAV and 48 hrs of GLPG0187 treatment on migratory capacity of UM-UC-3luc2 and RT-4 cells as determined by Transwell Boyden chamber migration assays. Mean numbers of migrated cells per area were measured. Mean number of migrated cells of the control NT cells are depicted above the respective bars (A). Effects of ITGAV knockdown and 48 hrs of GLPG0187 treatment on the CDH1/CDH2 ratio (B). Data were normalized to the NT or control conditions (n = 3) and are presented as mean ± SEM. qPCR (C) and protein analysis of SNAI1 (D). qPCR analysis (E) and protein analysis of SNAI2 (F). qPCR analysis (G) and protein analysis of ZEB1 (H). qPCR analysis (I) and protein analysis of ZEB2 (J). Relative expression levels are shown compared to respectively NT or vehicle treated cells. All qPCR values were normalized for GAPDH and presented as mean ± SEM.

Furthermore, ITGAV knockdown showed a significant and substantial decrease in migratory capacity in a Transwell Boyden chamber assay in both sh clones of UM-UC-3luc2 and RT-4 cells (Figure 2A). Accordingly, GLPG0187 treatment of UM-UC-3luc2 and RT-4 cells resulted in a dose-dependent and significant decrease in migration, and almost completely inhibited migration with the highest dosage used (Figure 2A).

### Effects of αv integrin on epithelial plasticity

Acquisition of an invasive phenotype is a requirement for metastasis where transformed epithelial cells can switch from a sessile, epithelial, to a motile, mesenchymal phenotype by epithelial- mesenchymal transition (EMT). Whether α_v_ integrin is functionally involved in the EMT-like switch in human bladder cancer has remained largely elusive. Therefore, we examined the effects on EMT of α_v_ knockdown or inhibition by GLPG0187 on the bladder cancer cells. The CDH1/CDH2 (E-cadherin/N-cadherin) ratio, was measured by flow cytometry. During EMT, epithelial markers, including CDH1, are downregulated and mesenchymal markers, including CDH2, are upregulated, resulting in a decrease of the CDH1/CDH2 ratio. Since EMT is believed to be required for the first steps in metastasis formation, the reverse process of MET, might be the mechanism behind the decreased migrative and clonogenic capacity following α_v_ kd and GLPG0187 treatment. As expected, CDH1 (E-cadherin) protein levels were relatively high in the epithelial-like RT-4 cells and negligible in the mesenchymal-shaped UM-UC-3 cells. CDH2 (N-cadherin) expression was higher in UM-UC-3 cells compared to RT-4 cells (Figure S2H–I). Hence, base line levels of the CDH1/CDH2 ratio were higher in the RT-4 cell line compared to the UM-UC-3luc2 cell line, corresponding to the more epithelial phenotype of the RT-4 cell line. CDH1/CDH2 ratio was significantly increased as a result of α_v_ kd in both cell lines (Figure 2B and Figure S2 A–I). CDH1 protein levels increased in the RT-4 cells and CDH2 protein levels significantly decreased in both cell lines (FACS plots shown in Figure S2A–B and S2H–I). GLPG0187 treatment showed a dose-dependent increase of the CDH1/CDH2 ratio which was significant in the highest dosages used in both UM-UC-3 and RT-4 cells (Figure 2B).

In line with our FACS analysis (Figure S2A), the expression of CDH1 is negligible in the UM-UC3 cells and displays a largely marginal increase in expression (green CDH1, blue DAPI staining of the nucleus, Figure S4A–C). CDH1 protein levels were below the detection limit of our western blot analysis in UM-UC-3 cells (data not shown). Membrane-expression of CDH1 in RT-4 cells increased in the ITGAV knockdown cells ((Figure S4G–H). CDH1 expression was also increased when RT-4 cells were treated with GLPG0187 (Figure S4I).

Vimentin levels were high in the UM-UC-3 cells, and remained similar in the ITGAV inhibited cells (Figure S4D–F), although the shape of the cells changed considerably. Vimentin levels in the RT-4 cells were markedly lower, and did not display changes upon inactivation of ITGAV. These data are in line with the FACS data using antibodies against CDH1 and Vimentin (respectively Figure S2A–B and S2F–G).

Subsequently, the mRNA and protein expression levels of EMT-inducing transcription factors were determined by real-time qPCR and Western Blot. The expression of SNAI1 was not changed in UM-UC-3 cells following α_v_ kd or treatment with GLPG0187 and was decreased in RT-4 cells with α_v_ kd knockdown and upon the GLPG0187 treatment (Figure 2C–D, S3A). SNAI2 mRNA and protein expression levels were significantly downregulated following α_v_ kd in both cell lines (respectively Figure 2E–F and S3B). Treatment with GLPG0187 showed a slight decrease in both mRNA and protein levels at the highest dosages used in UM-UC-3 cells (respectively Figure 2E–F and S3B). The effect of GLPG0187 on SNAI2 protein expression in RT-4 cells is very mild (Figure 2F).

Zeb1 mRNA was slightly decreased in UM-UC-3 cells upon knockdown and GLPG0187 treatment, and in certain concentrations of GLPG0187 in RT-4 cells (Figure 2G). Western Blot displayed a decrease in ZEB1 protein expression levels in both cell lines in the knockdown conditions (Figure 2H and S3C). GLPG0187 did not alter ZEB1 protein levels. It should be noted that the ZEB1 antibody possesses low specificity and therefore could represent an artefact (Figure S3D).

Zeb2 mRNA levels were significantly downregulated following α_v_ kd (Figure 2I). Treatment with GLPG0187 showed a slight decrease at the highest dosages used (Figure 2I). ZEB2 protein expression levels were measured with FACS analysis and demonstrated a significant decrease in ZEB2 protein expression in the sh clones in both cell lines (Figure 2J). In addition, the ZEB2 protein expression was dose-dependently decreased upon addition of GLPG0187 in both cell lines (Figure 2J). However, when measuring the protein levels with Western Blot analysis, we repeatedly found a variety of bands unfortunately rendering these experiments non-conclusive (Figure S3D). No significant effect was observed on the expression of TWIST mRNA expression in both cell lines (Figure S3I). In RT-4 cells no expression of TWIST was detected (Ct value>36; Figure S3I).

### Effects of αv integrin on clonogenicity, stemness and metastasis markers

**Figure 3:**
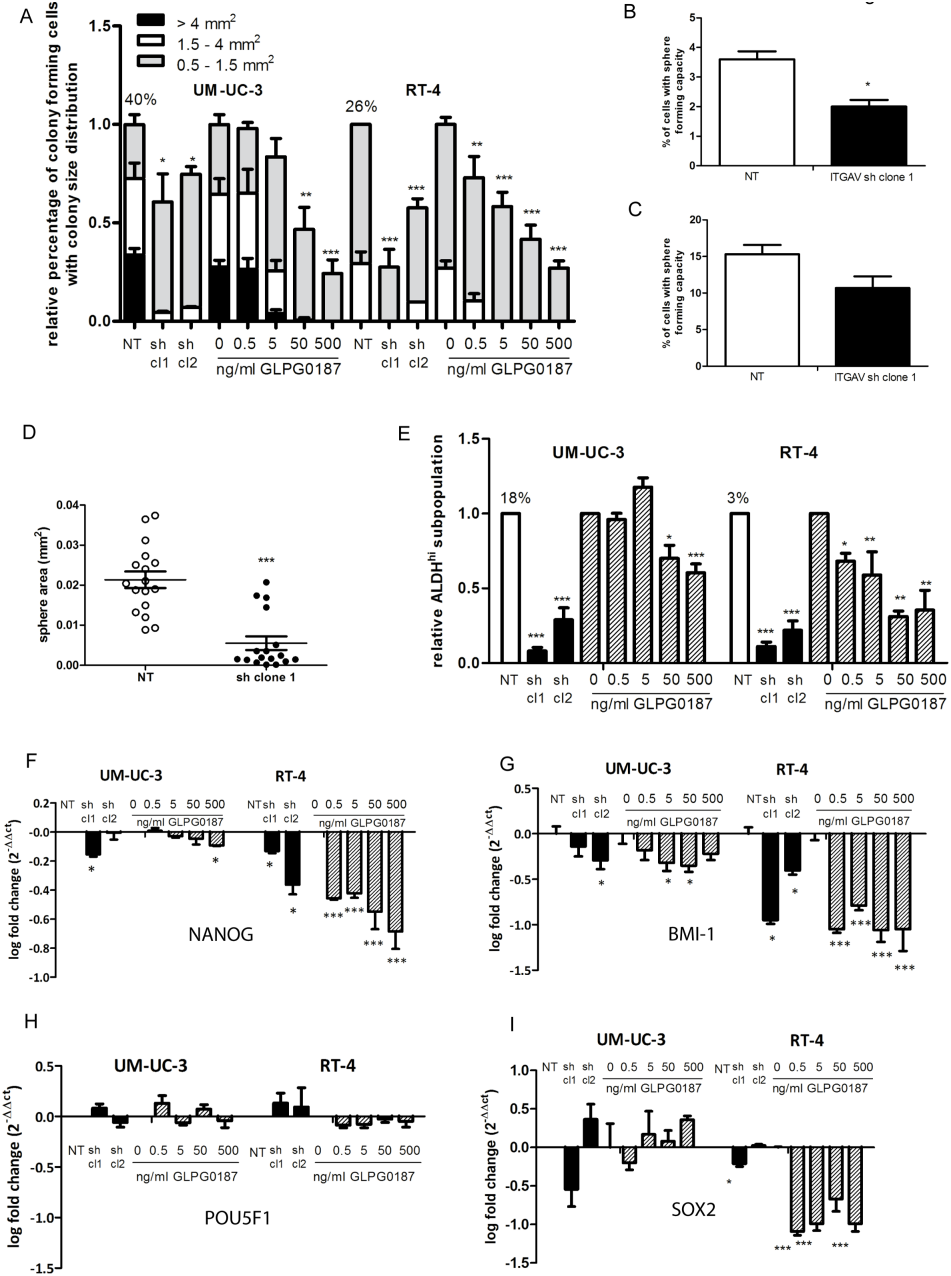
Effects of α_v_ integrin on clonogenicity and stem cell/metastasis markers. The relative percentage and size distribution of colony-forming cells in a 96-wells plate clonogenic assay of single-cell diluted cultures after 2 weeks in the α_v_ kd or NT cells and cells treated with a dose range of GLPG0187 for 48 hrs and plated afterwards. The area of the colonies was measured with Image J software and divided according to size. Small colonies are between 0.5 and 1.5 mm^2^, medium sized colonies are between 1.5 and 4 mm^2^ and large colonies are bigger than 4 mm^2^. Data were normalized to the NT or control conditions and are presented as mean ± SEM. Percentage of colony-forming cells in the control NT cells are depicted above the respective bars (A). UM-UC-3 cells were seeded 100 cells/cm^2^ in an ultra-low attachment plate in serum-starved conditions. The percentage of cells with sphere forming capacity (P0) was measured after 10 days of culture (B). P0 spheres were dissociated into single cells and seeded in ultra-low attachment 96 wells. The percentage of cells with sphere forming capacity (P1) was measured after 10 days of culture (B). The area of the spheres was measured with Image J software (D). Percentage of cells with high ALDH activity (ALDH^hi^) as measured with Aldefluor assay. Data are normalized to the NT or vehicle treated cells. Percentages of ALDH^hi^ cells in the NT and control cells are depicted above the respective bars (E). qPCR analysis of NANOG (F) qPCR analysis of BMI1 (G). Relative expression levels are shown compared to respectively NT or vehicle-treated cells. All values were normalized for GAPDH and presented as mean ± SEM.

Next, we investigated and compared the effect of α_v_ knockdown on clonogenicity and previously identified bladder cancer stem cell markers. Compared to the UM-UC-3 cell line, RT-4 cell line produced less and smaller colonies in a single cell colony-forming assay, in line with the less malignant phenotype of this cell line. As shown in figure 3A, ITGAV knockdown significantly reduced the clonogenic capacity of both cell lines. Furthermore, the size of the colonies was significantly decreased upon α_v_ knockdown. Addition of GLPG0187 significantly reduced the clonogenic capacity as well as the size of the colonies in both cell lines (Figure 3A and S5A).

Previously, sphere formation has been described as a characteristic of CSCs that reflects the potential for self-renewal. We have identified a subpopulation of cells with urosphere forming ability within the UM-UC-3 cells. After 10 days of culture in ultra-low attachment plates using serum-starved culture conditions, p0 urospheres were counted (schematic representation in Figure S5B) [35]. The efficiency of urosphere formation (P0) varied and was highest in UM-UC-3 NT cells compared to the cells with ITGAV knockdown ((3.6±0.87% for the UM-UC-3luc2 NT cells versus 2.0±0.72% for the UM-UC-3 ITGAV sh clone1, Figure 3B). In addition, the size of the urospheres was significantly higher in the NT cells compared to the ITGAV knockdown cells (Figure 3D). Furthermore, the self-renewing capacity of these cells was measured by dissociating the obtained primary P0 spheres into single cells and plating one cell per well in anchorage-independent, serum-starved conditions. After 10 days, respectively 15,3% and 10,7% secondary spheres were formed in the (P1) from UM-UC-3 NT cells compared to the cells with ITGAV knockdown. It was also possible to generate further new spheres from single-cell suspension of P1 urospheres (Figure 3C). This clonal self-renewal could be observed for at least 3 passages. These data indicate that UM-UC-3luc2 cells are able to form urospheres, which could be propagated for several passages. ITGAV knockdown decreased the ability of the cells to form urospheres, the size of the formed spheres, and, in addition, decreased the capacity to form secondary spheres.

High ALDH activity (ALDH^hi^) has recently been proposed to be indicative of cancer stem/progenitor cells in bladder cancer [25]. In line with this, the size of the ALDH^hi^ subpopulation of was significantly smaller in the RT-4 cells compared to the more malignant UM-UC-3luc2 cells (Figure 3E). Both ITGAV knockdown and GLPG0187 treatment significantly decreased the ALDH^hi^ subpopulation in both the UM-UC-3luc2 and RT-4 cells. This effect was most prominent in RT-4 cells (Figure 3E).

Then, the expression of previously identified bladder cancer stem cell markers (POU5F1, BMI1, NANOG, SOX2,) was determined by real- time qPCR in the established control NT and αv kd cell lines as well as in GLPG0187 treated cells. The expression of NANOG and BMI1 was significantly decreased upon ITGAV knockdown and GLPG treatment (Figure 3F–G). The expression of POU5F1 was not affected by ITGAV knockdown or GLPG0187 treatment in either cell line (Figure 3H), whereas the expression of SOX2 only decreased in the RT-4 cells (Figure 3I).

**Figure 4:**
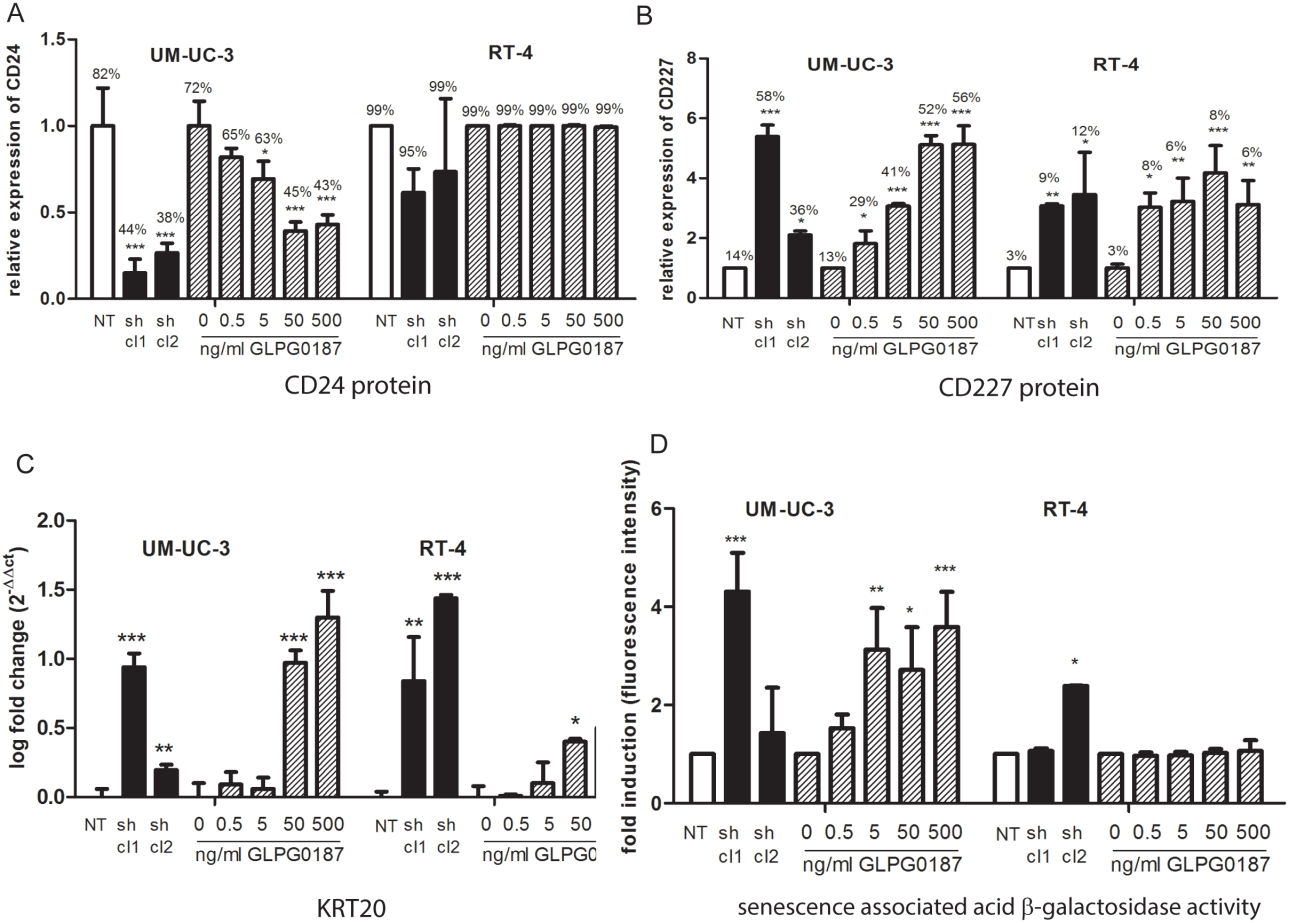
Effect of α_v_ integrin on expression levels of CD24 and urothelial differentiation markers and on senescence. Relative expression levels of CD24 (A) and CD227 (B). Relative expression levels (% of positive cells * Mean fluorescence intensity) were measured by flow cytometry and normalized to the NT or vehicle treated cells. Data are represented as mean ± SEM. Percentages of positive cells are depicted above the respective bars. qPCR analysis of KRT20. Relative expression levels are shown compared to respectively NT or non-treated cells. All values were normalized for GAPDH and presented as mean ± SEM (C). UM-UC-3 luc2 and RT-4 cells were seeded into a six-well plate and exposed to a concentration series of GLPG0187 (0–500 ng/ml). 48 h after incubation, cells were harvested and senescence associated acid β-galactosidase activity was measured. Data are represented as fold change in fluorescence intensity of the signal (D).

Recently, CD24 has been described as a marker for bladder cancer metastasis formation and was shown to be required for metastasis to the lungs [36]. Interestingly, CD24 protein expression levels were significantly decreased by approximately 80% in α_v_ kd UM-UC-3 cells, whereas no effect on CD24 expression was observed in α_v_ kd RT-4 clones (Figure 4A and S6O and S6P). GLPG0187 treatment resulted in a dose-dependent decrease in CD24 expression in UM-UC-3 cells (Figure 4A). Similar to the α_v_ kd, GLPG0187 treatment did not affect CD24 expression in RT-4 cells (Figure 4A).

No effects were observed on the protein expression of other putative bladder cancer stem cells, e.g. CD133 and CD44v6, by ITGAV knock down or GLPG0187 treatment in either cell line (Figure S6G–K). CD44 protein expression levels slightly decreased, especially in the RT4 cells with ITGAV knockdown (Figure S6L–N).

The expression of CD227 (Mucin-1), a negative marker for basal cells, increased by ITGAV knockdown or treatment with GLPG0187 (dose-dependently) (Figure 4B and S6Q–R). In addition, the expression of KRT20, a keratin which is expressed in differentiated umbrella cells, but not in basal cells or intermediate cells was significantly increased upon ITGAV knockdown or treatment with high doses of GLPG0187 (Figure 4C).

It is important to note that although base line levels of both the migratory and clonogenic capacity of RT-4 cells are much lower than those of UM-UC-3luc2 cells, the percentage of reduction is similar in both cell lines (Figure 2 and 3).

In addition to the effects on stemness and epithelial plasticity, functional inactivation of ITGAV by blocking the ITGAV receptor using GLPG0187, dose-dependently induced cellular senescence in UM-UC-3 cells as measured by acidic senescence associated β-galactosidase activity (Figure 4D). ITGAV knockdown resulted in increased senescence in only one of the two short hairpin clones in both cell lines (Figure 4D).

Taken together, both α_v_ kd and GLPG0187 treatment of UM-UC-3luc2 and RT-4 cell lines, were able to reduce several malignant characteristics *in vitro*.

### Effects of αv integrin on tumor growth and metastasis in preclinical *in vivo* models

**Figure 5:**
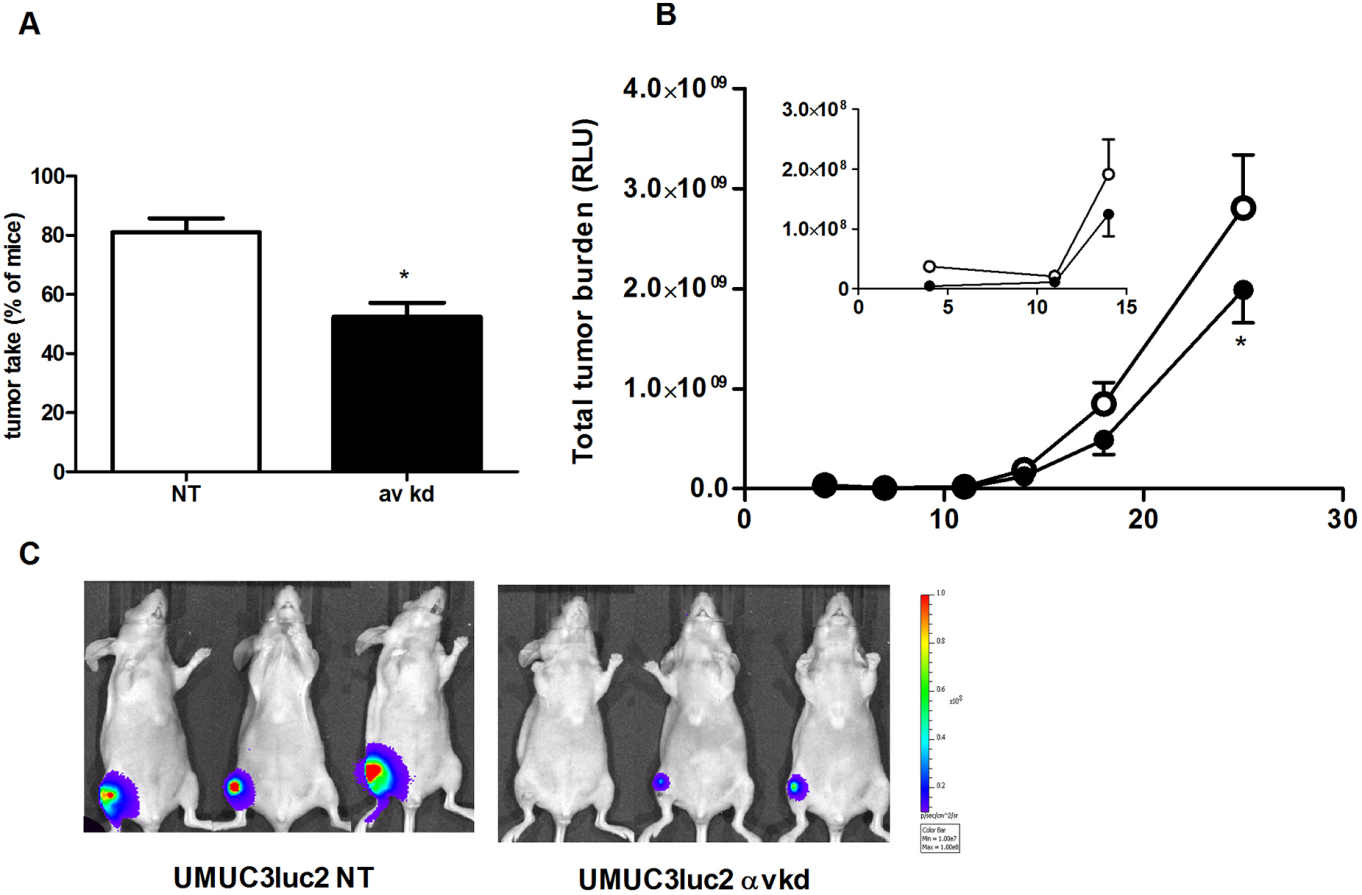
ITGAV knockdown in UM-UC-3luc2 cells affects intra-bone growth in a preclinical model. A) Percentage of mice with tumors after intra-bone inoculation of either α_v_-kd-UM-UC-3luc2 or NT-UM-UC-3luc2 cells B) Total tumor burden of the mice injected with α_v_-kd-UM-UC-3luc2 (*closed circles*) or NT-UM-UC-3luc2 cells (*open circles*). C) Representative images of mice intra-osseously inoculated with either αv-kd-UM-UC-3luc2 or NT-UM-UC-3luc2 cells 7 days after inoculation (*n* = 10/group *P<0.05).

Subsequently, we analyzed and compared the tumorigenicity and metastatic ability of the αv kd UM-UC-3luc2 cells *in vivo*. We investigated the capacity of the cells to grow in bone marrow, one of the sites of bladder cancer metastasis. The amount of mice displaying metastatic foci, as measured with sensitive bioluminescent imaging of the UMUC3 luciferase 2 cells in real-time after inoculation of the αv kd UM-UC-3luc2 cells in the bone marrow, was strikingly lower compared to the NT cell population (Figure 5A). Moreover, we observed significantly decreased total tumor burden in the mice injected with αv kd cells compared to the mice injected with the NT (Figure 5B–C).

Metastasis formation was significantly decreased in mice inoculated intra-cardiacally with 10,000 αv kd cells vs. NT cells (Figure S7A) [34]. Total tumor burden as well as bone tumor burden was decreased upon ITGAV kd, albeit not significantly (Figure S7B–D).

**Figure 6:**
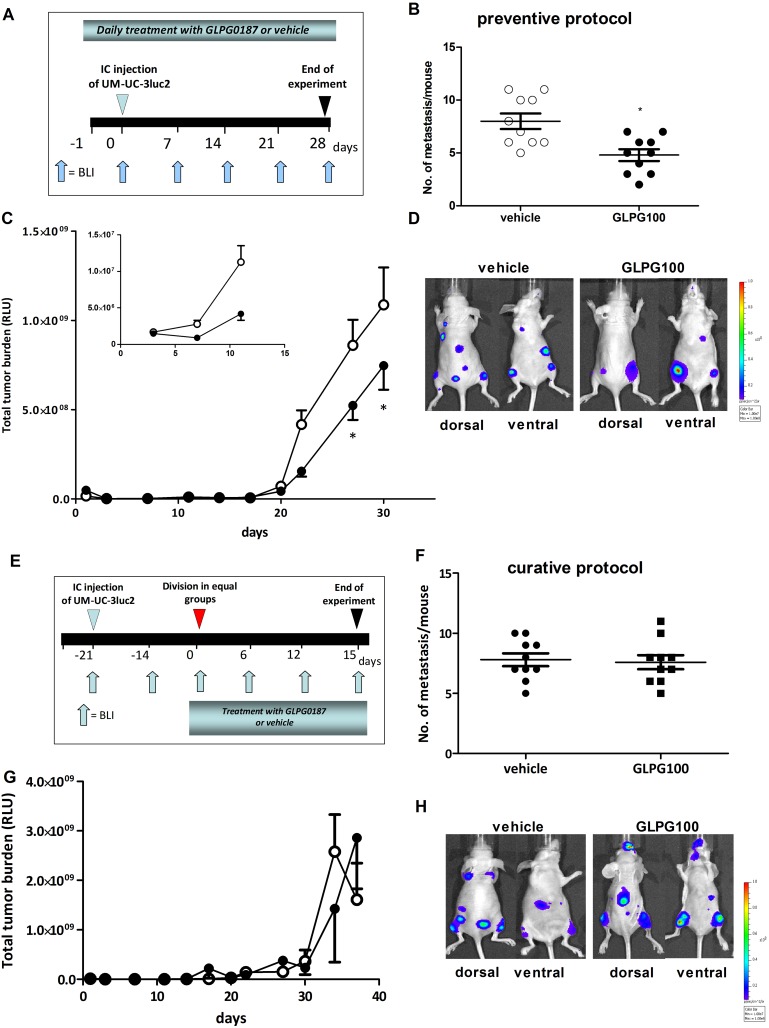
Effect of systemic administration of GLPG0187 on tumor growth and metastasis in a preventive and curative protocol. A) Schematic representation of the *preventive* protocol. Mice were treated daily with either IP administrated vehicle or GLPG0187 (100 mg/kg/day) from day -1 onwards. At day 0, 100,000 UM-UC-3luc2 cells were inoculated into the left heart ventricle and once a week BLI images were taken. B) Number of metastasis per mouse. C) Total tumor burden for the mice treated with 100 mg/kg/day GLPG0187 (closed circles) or vehicle (open circles). In the insert, the first 14 days are shown. D) Representative images of mice treated with vehicle or 100 mg/kg/day GLPG0187 taken at day 28 after inoculation. E) Schematic representation of the *curative* protocol. At day -21, 100,000 UM-UC-3luc2 cells were injected into the left heart ventricle and once a week BLI images were taken. At day 0, mice were divided into groups with equal total tumor burden. Mice were daily treated with an IP dosage of either vehicle or GLPG0187 (100 mg/kg/day) from day 0 onwards. F) Number of metastasis per mouse. G) Total tumor burden for the mice treated with 100 mg/kg/day GLPG0187 (closed circles) or vehicle (open circles). H) Representative images of mice treated with vehicle or 100 mg/kg/day GLPG0187 taken at day 15 after start of treatment.

Next, we investigated whether blocking α_v_-integrin can be used to treat experimentally-induced bone metastasis from intracardiacally inoculated UM-UC-3luc2 cells according to a *preventive* protocol (Figure 6A). The number of metastasis (Figure 6B) as well as the total tumor burden (Figure 6C) was significantly decreased in mice daily treated with 100 mg/kg/day GLPG0187 (IP).

In the *curative* protocol, bone metastases were allowed to develop for 21 days (Figure 6E). Subsequently, the mice were treated with a daily dosage of either vehicle or GLPG0187 (100 mg/kg/d IP) to investigate whether blocking α_v_-integrin can be used to treat already existing bone metastases. However, no significant effects were found on the amount of metastasis/mouse, total tumor burden or bone tumor burden after 15 days of treatment with 100 mg/kg/d GLPG0187 (Figure 6F–H).

Taken together, knockdown or treatment of α_v_ integrins resulted in decreased metastatic growth in preclinical *in vivo* models as assessed by BLI.

## Discussion

Low survival rates of metastatic bladder cancer emphasize the need for a drug that can prevent and/or treat metastatic cancer. A promising approach that has been explored for these means in breast [29], melanoma [30] and prostate [37] cancer is the targeting of α_v_ integrins, which has been shown to reduce tumor growth, metastasis and angiogenesis.

In this study, the role of ITGAV, which are highly expressed in bladder carcinomas, and its potential as a drug target in bladder cancer were investigated both by treatment with the ITGAV integrin-inhibitor GLPG0187 and knockdown of ITGAV. RT-4 and UM-UC-3luc2 cells were investigated to determine whether α_v_ integrin targeted therapies could be beneficial for these different grades of bladder tumors. The rationale for targeting of ITGAV is their involvement in cell proliferation, migration, invasion, survival and angiogenesis, which are essential processes for primary tumor growth and metastasis formation [26], [38], [39]. These processes are induced via activation of focal adhesion kinase (PTK2) and src-family kinases (SFKs), that activate the ERK/MAPK, NF-κB and AKT/PKB pathways [40]. Modulating adherence to the extracellular matrix (ECM) by changing the affinity of integrins for their ECM ligands, is important for the motility of cancer cells [41].

Both GLPG0187 treatment and knockdown of ITGAV resulted in loss of adhesion, resembling loss of adherence to the ECM, which might have an inhibitory effect on cell motility and migration. Indeed, a strong decrease in migratory capacity was found after both ITGAV knockdown and GLPG0187 treatment, indicating a less malignant phenotype. However, especially in the knockdown sh clones, we cannot exclude that decreased adhesion and proliferation plays a role in the decreased migratory ability.

Reduced proliferation and clonogenicity upon α_v_ integrin inactivation provided further evidence for this reduced malignancy. In line with the functional differences, α_v_ kd and treatment with GLPG0187 resulted in a more epithelial phenotype, as illustrated by an increased CDH1/CDH2 ratio. This indicates that cells have undergone at least partial MET and thus that α_v_ integrins might be important for the maintenance of a mesenchymal phenotype (EMT). EMT can be regulated by transcription factors including SNAI1, SNAI2, TWIST, ZEB1 and ZEB2, that directly or indirectly repress CDH1 expression, which is considered to be a fundamental event in EMT [42]. In bladder cancer, SNAI1, SNAI2 and TWIST are differentially expressed [43] and TWIST and SNAI2 expression have been shown to play an important role in tumor progression and metastasis formation [44]–[47]. Since knockdown of ITGAV and to a lesser extent treatment with the GLPG0187 compound in UM-UC-3 and RT-4 cells resulted in a significant decrease in SNAI2, SNAI2 might play an important role in EMT in bladder cancer. The expression of ZEB1 and 2 also decreased, however, this is not conclusive since the ZEB antibodies possess low specificity.

Recent evidence suggests that EMT generates cells with stem/progenitor-like properties and enables plasticity between CSC and non-CSC [4], [7]–[9]. Therefore, EMT and CSC populations might show a large overlap [48], [49]. Furthermore, EMT plays a critical role not only in invasion and metastasis but also in tumor recurrence that is believed to be tightly linked with the biology of cancer stem/progenitor cells. Indeed, the clonogenic capacity significantly decreased upon knockdown of ITGAV or treatment with GLPG0187. In line with these data, ITGAV knockdown and GLPG0187 treatment demonstrated a slight, but significant decrease in the expression of NANOG and BMI1, which have been shown to be regulators of self-renewal in embryonic stem cells [50]–[52]. Another self-renewal marker, SOX2, was only downregulated in RT-4 cells. In addition, α_v_ kd and GLPG0187 treatment resulted in a decreased percentage of cells with high ALDH activity. This subpopulation has recently been shown to be involved in stemness and metastasis formation in several solid cancers, including breast [22], ovarian [23], prostate [24] and bladder [25] cancer. A possibility is that α_v_ integrin functions upstream of ALDH and that the reduction in ALDH activity is responsible for the decreased migratory potential after α_v_ kd and GLPG0187 treatment.

In this study, α_v_ kd and GLPG0187 treatment resulted in decreased urosphere area (P0) and decreased self-renewal ability, as assessed with the urosphere assay. However, no effects were found on the mRNA expression levels of POU5F1 (Oct-4), which has been proposed to be a key regulator for pluripotency and self-renewal in embryonic stem cells [53]. POU5F1 has also been shown to have an important role in bladder cancer stem cells, invasion and migration [15], [54]–[56], however, our data suggest that α_v_ integrin might affect migration through a mechanism independent of POU5F1, for example via NANOG or SOX2. Alternatively, α_v_ integrin might function downstream of POU5F1.

The decrease in stemness genes coincides with an increase in CD227, a negative marker for basal cells, indicating increased levels of differentiation upon α_v_ kd and GLPG0187 treatment.

CD24 has recently been proposed as a drug target for anti-metastatic therapy in bladder cancer [36]. The level of expression was shown to be increased in human bladder cancer metastasis compared to primary tumor. In addition, CD24 expression has been correlated with increased metastasis formation of UM-UC-3 cells in a lung metastasis mouse model [36]. In this study, we showed a decrease in CD24 expression in UM-UC-3luc2 cells upon α_v_ kd and GLPG0187 treatment. Interestingly, acidic senescence associated β-galactosidase activity increased upon functional inactivation of ITGAV, indicating that inhibition of ITGAV increases the amount of senescent cells. Senescent cells do not enter the mitotic cycle even in the presence of growth factors, they are however alive and remain metabolically active. Activating the senescence cell cycle arrest provides a tumor suppressor mechanism that can inhibit cancer growth as shown previously in multiple tumors.

In conclusion, the effects observed with α_v_ kd and GLPG0187 treatment in both cell lines show that ITGAV inhibition results in a less malignant phenotype *in vitro.* α_v_ integrin appears to be involved in proliferation, migration and clonogenicity through EMT induction, the maintenance of cancer stem/progenitor-like ALDH^hi^ cells and CD24 expression. In line with the *in vitro* observations, knockdown of ITGAV or treatment with GLPG0187 significantly inhibited metastasis and secondary tumor growth *in vivo*. One of the models used, the experimental metastasis model, displays the effects of the tumor cells injected into the left cardiac ventricle that subsequently metastasize to the secondary target organs. In this model, the cells have to survive in the bloodstream, extravasate and colonize secondary target organs. The other xenograft transplantation model involves inoculation at the secondary target organ (in this case the bone microenvironment), using this model, the cells have to survive and grow at the secondary site.These data indicate, that in addition to the effects on angiogenesis and osteoclastogenesis [27], GLPG0187 also affects colonization of the metastatic sites and tumor growth.

The effects of GLPG0187 on tumor growth in the curative protocol might be improved by combination therapy with commonly used chemotherapeutics that will target the more differentiated cells in established tumors.

Taken together, we show for the first time that targeting of α_v_ integrin appears to be a promising approach for treatment and/or prevention of metastatic bladder cancer. Further research should help to elucidate whether GLPG0187 would be beneficial in combination with established therapeutic interventions.

## Supporting Information

Figure S1 **Expression of ITGAV, effect on viability and proliferation.** Real time qPCR analysis of ITGAV (A). Values were normalized for GAPDH and presented as mean ± SEM. Relative expression levels are shown compared to NT cells. Representative images of flow cytometry plots of relative ITGAV expression levels in UM-UC-3luc2 (B) and RT4 (C) transduced with an shRNAi construct targeting ITGAV (sh clone1 and 2) or a non-targeting short hairpin (NT). Tissue sections of normal human bladder urothelium (D) and human bladder carcinoma (E) stained with α_v_ integrin antibody (data source: www.proteinatlas.org, Novacastra). Relative expression of ITGAV protein expression in NT controls cells (open bars), floating ITGAV knockdown cells (black bars) and in ITGAV knockdown cells that are still attached to the tissue culture plastic (striped bars). At the x-axis, the % of cells with the phenotype is depicted (F). The amount of viable, apoptotic and dead cells in the α_v_ kd and NT UM-UC-3 and RT-4 cells were measured using the Alexa Fluor 488 annexin V/Dead Cell Apoptosis Kit (Invitrogen). In addition, UM-UC-3 luc2 and RT-4 cells were seeded into a 6-well plate and exposed to a concentration series of GLPG0187 (0–500 ng/ml). 48 h after incubation, cells were harvested and processed for annexin V/PI staining. The percentage of viable (AnnexinV−/PI−), dead (PI+/AnnexinV−), and total apoptotic cells (AnnexinV+) are shown (G). Proliferation rate (mitochondrial activity as assessed with 3-(4,5-dimethylthiazol-2-yl)-2,5-diphenyltetrazolium bromide (optical density at 490 nm)) in the 2 α_v_ kd clones (respectively closed circles and triangles) and NT (open circles) UM-UC3luc2 (H) and RT-4 (I) cells. The effects of GLPG0187 treatment on proliferation rate of UM-UC-3luc2 (J) and RT-4 cells (K) after 24, 48 and 72 h of treatment was assessed with 3-(4,5-dimethylthiazol-2-yl)-2,5-diphenyltetrazolium bromide (optical density at 490 nm). Data are presented as mean ± SEM (n = 3).
(TIF)

Figure S2 **Protein levels of EMT markers.** Representative images of flow cytometry plots of relative E-cadherin expression levels in UM-UC-3luc2 (A) and RT-4 (B) cells transduced with an shRNAi construct targeting ITGAV (sh clone1 and 2) or a non-targeting short hairpin (NT). Western Blot analysis of E-cadherin and b-actin in RT-4 cells (C) and densitometry analysis of the relative protein expression levels, measured with western blot analysis, compared to respectively NT or vehicle treated cells and corrected for b-actin expression levels (D). Representative images of flow cytometry plots of relative Vimentin expression levels in UM-UC-3luc2 (F) and RT-4 (G) cells transduced with an shRNAi construct targeting ITGAV (sh clone1 and 2) or a non-targeting short hairpin (NT). Representative images of flow cytometry plots of relative N-cadherin expression levels in UM-UC-3luc2 (H) and RT-4 (I) cells transduced with an shRNAi construct targeting ITGAV (sh clone1 and 2) or a non-targeting short hairpin (NT).
(TIF)

Figure S3 **Protein levels of intracellular EMT markers.** Densitometry analysis of the relative protein expression levels of SNAI1 (A), SNAI2 (B) and ZEB1 (C), measured with western blot analysis, compared to respectively NT or vehicle treated cells and corrected for b-actin expression levels in UM-UC-3 cells or RT-4 cells (respectively NT, sh clone 1, control and a concentration series of GLPG0187). Whole audiograms of ZEB1 and ZEB2 western blot analysis, displaying multiple additional bands (D). Representative images of cytometry plots of ZEB2 protein expression in UM-UC-3 NT and sh clones 1 and 2 (E) and ZEB2 protein expression in RT-4 NT and sh clones 1 and 2 (F). Representative images of cytometry plots of ZEB2 protein expression in UM-UC-3 cells (G) or RT-4 cells (H) treated with a dose-range of GLPG0187. Real time qPCR analysis of TWIST in UM-UC-3 and RT-4 cells (I). Relative expression levels are shown compared to respectively NT or non-treated cells.
(TIF)

Figure S4 **Immunofluorescence of E-cadherin and Vimentin.** Representative confocal images of E-cadherin staining in UM-UC-3 NT (A), ITGAV knockdown clone 1 (B) and UM-UC-3 cells treated with 500 ng/ml GLPG0187 for 24 h (C) Representative confocal images of Vimentin staining in UM-UC-3 NT (D), ITGAV knockdown clone 1 (E) and UM-UC-3 cells treated with 500 ng/ml GLPG0187 for 24 h (F). Representative confocal images of E-cadherin staining in RT-4 NT (G), ITGAV knockdown clone 1 (H) and UM-UC-3 cells treated with 500 ng/ml GLPG0187 for 24 h (I) Representative confocal images of Vimentin staining in RT-4 NT (J), ITGAV knockdown clone 1 (K) and RT-4 cells treated with 500 ng/ml GLPG0187 for 24 h (L).
(TIF)

Figure S5 **Tumor-initiating cell characteristics.** Representative image of a colony in a clonogenic assay of UM-UC-3 cells 14 days after seeding (5x magnification) (A). Schematic representation of the urosphere protocol, adapted from Bisson et al [35]. (B) Representative images of UM-UC-3 NT (C) and ITGAV knockdown (D) P0 urospheres 10 days after seeding. Scale bar represents 50 µm (20x magnification).
(TIF)

Figure S6 **Expression levels of markers.** Expression levels of ITGAV knockdown clones 1 and 2 were compared to control cells transduced with a non-targeting short hairpin (NT). Cells were treated with GLPG0187 concentration series for 48 h. Data were normalized to the NT or control conditions and are presented as mean ± SEM. Relative expression levels of ITGA2-FITC (percentage of positive cells * mean fluorescence intensity (A) with representative cytometry plot for UM-UC-3 (B) or RT-4 cells (C). Relative expression levels of ITGA6-APC (percentage of positive cells * mean fluorescence intensity (D) with representative cytometry plot for UM-UC-3 (E) or RT-4 cells (F). Relative expression levels of CD133-APC (percentage of positive cells * mean fluorescence intensity (G) with representative cytometry plot for UM-UC-3 (H). Relative expression levels of CD44v6-FITC (percentage of positive cells * mean fluorescence intensity (I) with representative cytometry plot for UM-UC-3 (J) or RT-4 cells (K). Relative expression levels of CD44v6-PE (percentage of positive cells * mean fluorescence intensity (L) with representative cytometry plot for UM-UC-3 (M) or RT-4 cells (N). Representative cytometry plot for CD24 expression in UM-UC-3 (O) or RT-4 cells (P). Representative cytometry plot for CD227 expression in UM-UC-3 (Q) or RT-4 cells (R).
(TIF)

Figure S7 **ITGAV knockdown in UM-UC-3luc2 cells affects metastatic growth in a preclinical model.** A) Number of metastases/mouse after intra-cardiac inoculation of either α_v_-kd-UM-UC-3luc2 or NT-UM-UC-3luc2 cells. B) Total tumor burden and C) tumor burden in long bones after intra-cardiac inoculation of either α_v_-kd-UM-UC-3luc2 (closed circles) or NT-UM-UC-3luc2 cells (open circles) D) representative images of mice at day 34 after inoculation.
(JPG)

Table S1 **Short hairpin RNAi constructs.** UM-UC-3luc2 and RT-4 cell lines were transduced with short hairpin RNAi constructs against ITGAV or scrambled non-targeting (NT) shRNA derived from Sigma’s MISSION library.
(DOC)

Table S2 **Antibodies with application, supplier and location.**
(DOCX)

Table S3 **Exon-spanning real-time PCR primers.** Exon-spanning real-time PCR primers were designed with Primer Express software (Applied Biosystems, Rotkreuz, Switzerland). KRT20 expression was measured with Taqman primer/probe set Hs00300643_m1 from Life Technologies.
(DOC)

Materials and Methods S1 **Materials and methods describing Annexin V/Propidium Iodide Apoptosis Assay, proliferation assay and Immunofluorescence staining.**
(DOC)
