# Supplementary figures and images for: Targeting of Alpha-V Integrins Reduces Malignancy of Bladder Carcinoma
Source: PLoS One. 2014 Sep 23;9(9):e108464. doi: 10.1371/journal.pone.0108464 (PMC4172769; doi:10.1371/journal.pone.0108464)

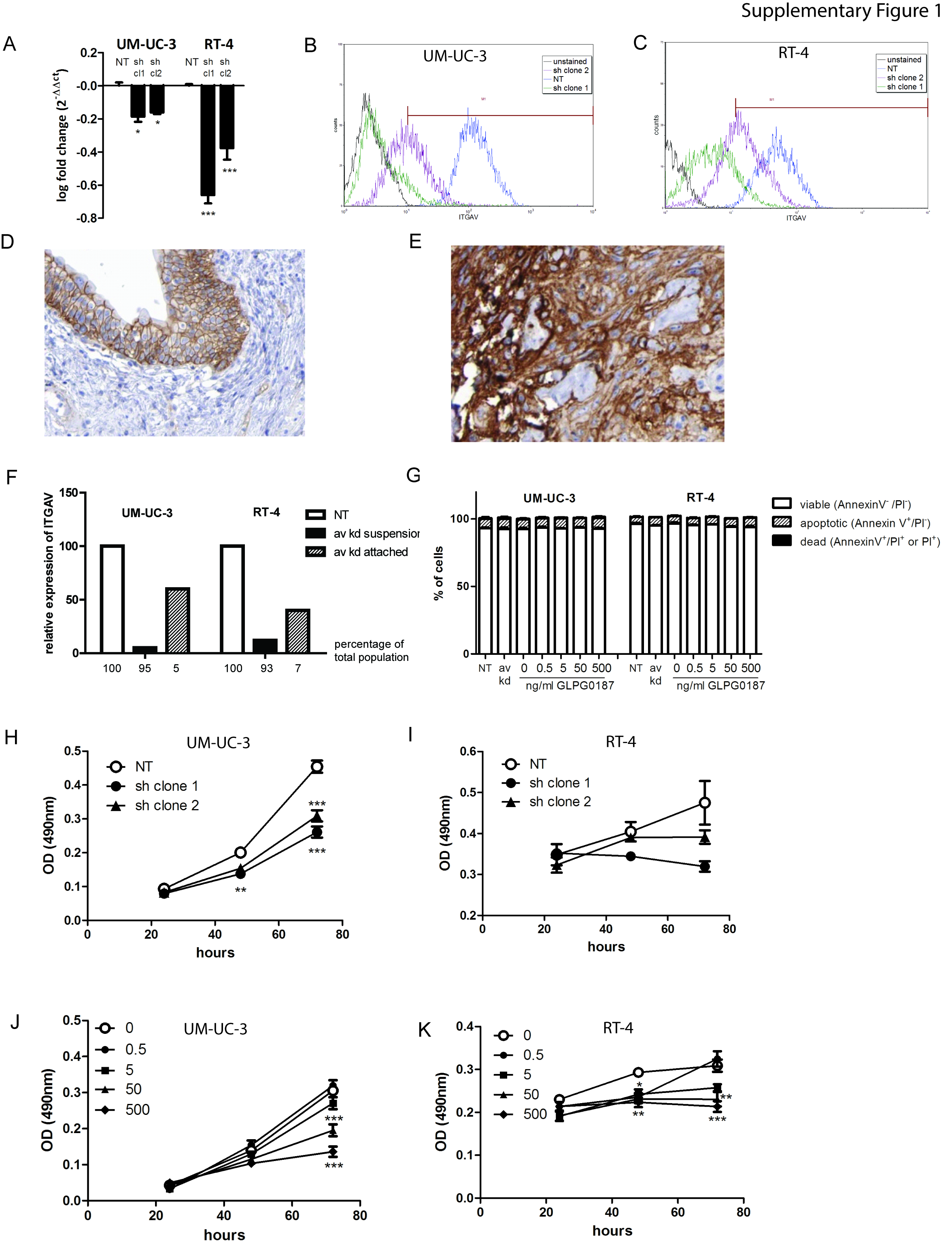

Supplement: Figure S1 — Expression of ITGAV, effect on viability and proliferation. Real time qPCR analysis of ITGAV (A). Values were normalized for GAPDH and presented as mean ± SEM. Relative expression levels are shown compared to NT cells. Representative images of flow cytometry plots of relative ITGAV expression levels in UM-UC-3luc2 (B) and RT4 (C) transduced with an shRNAi construct targeting ITGAV (sh clone1 and 2) or a non-targeting short hairpin (NT). Tissue sections of normal human bladder urothelium (D) and human bladder carcinoma (E) stained with αv integrin antibody (data source: www.proteinatlas.org, Novacastra). Relative expression of ITGAV protein expression in NT controls cells (open bars), floating ITGAV knockdown cells (black bars) and in ITGAV knockdown cells that are still attached to the tissue culture plastic (striped bars). At the x-axis, the % of cells with the phenotype is depicted (F). The amount of viable, apoptotic and dead cells in the αv kd and NT UM-UC-3 and RT-4 cells were measured using the Alexa Fluor 488 annexin V/Dead Cell Apoptosis Kit (Invitrogen). In addition, UM-UC-3 luc2 and RT-4 cells were seeded into a 6-well plate and exposed to a concentration series of GLPG0187 (0–500 ng/ml). 48 h after incubation, cells were harvested and processed for annexin V/PI staining. The percentage of viable (AnnexinV−/PI−), dead (PI+/AnnexinV−), and total apoptotic cells (AnnexinV+) are shown (G). Proliferation rate (mitochondrial activity as assessed with 3-(4,5-dimethylthiazol-2-yl)-2,5-diphenyltetrazolium bromide (optical density at 490 nm)) in the 2 αv kd clones (respectively closed circles and triangles) and NT (open circles) UM-UC3luc2 (H) and RT-4 (I) cells. The effects of GLPG0187 treatment on proliferation rate of UM-UC-3luc2 (J) and RT-4 cells (K) after 24, 48 and 72 h of treatment was assessed with 3-(4,5-dimethylthiazol-2-yl)-2,5-diphenyltetrazolium bromide (optical density at 490 nm). Data are presented as mean ± SEM (n = 3). (TIF) [file pone.0108464.s001.tif]

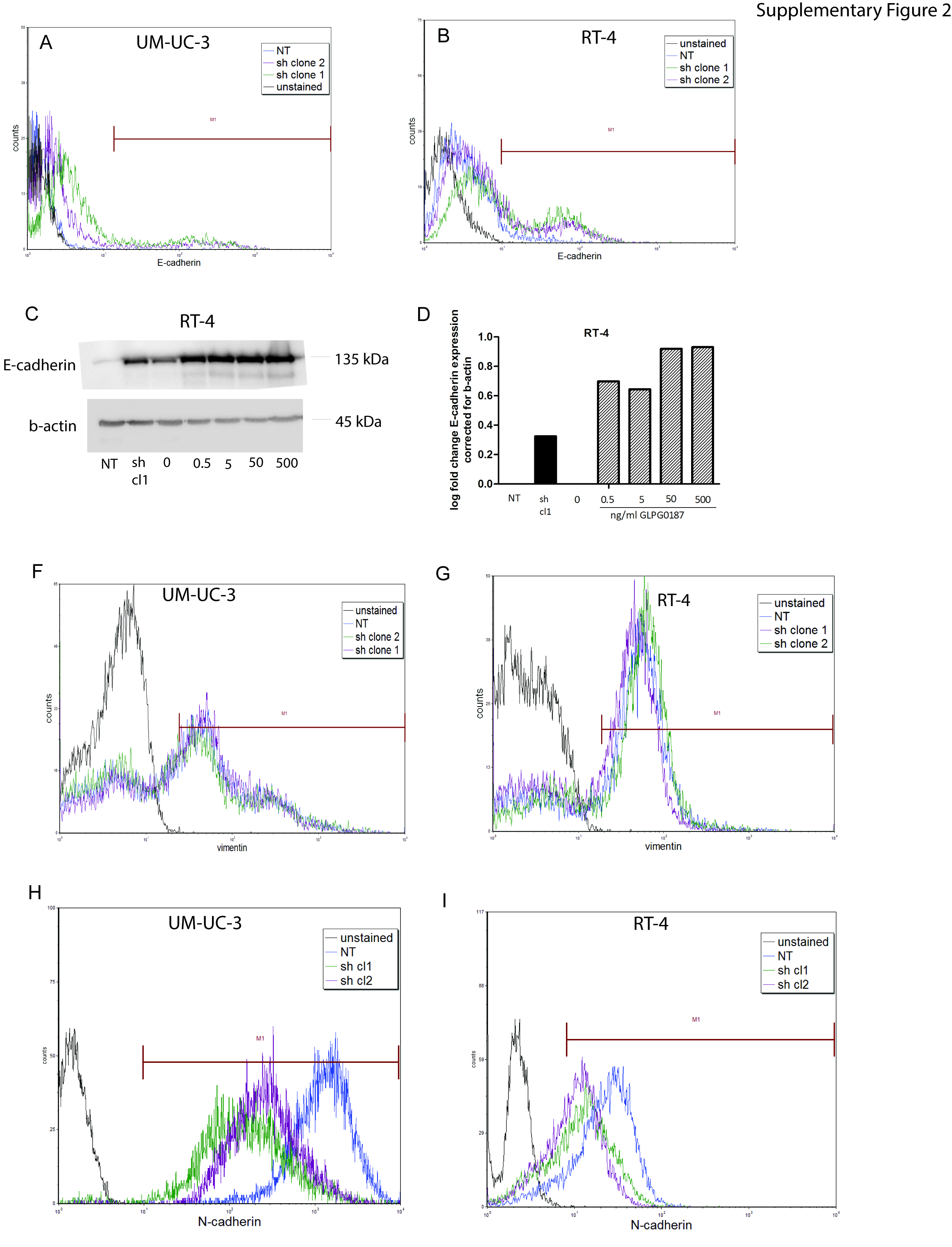

Supplement: Figure S2 — Protein levels of EMT markers. Representative images of flow cytometry plots of relative E-cadherin expression levels in UM-UC-3luc2 (A) and RT-4 (B) cells transduced with an shRNAi construct targeting ITGAV (sh clone1 and 2) or a non-targeting short hairpin (NT). Western Blot analysis of E-cadherin and b-actin in RT-4 cells (C) and densitometry analysis of the relative protein expression levels, measured with western blot analysis, compared to respectively NT or vehicle treated cells and corrected for b-actin expression levels (D). Representative images of flow cytometry plots of relative Vimentin expression levels in UM-UC-3luc2 (F) and RT-4 (G) cells transduced with an shRNAi construct targeting ITGAV (sh clone1 and 2) or a non-targeting short hairpin (NT). Representative images of flow cytometry plots of relative N-cadherin expression levels in UM-UC-3luc2 (H) and RT-4 (I) cells transduced with an shRNAi construct targeting ITGAV (sh clone1 and 2) or a non-targeting short hairpin (NT). (TIF) [file pone.0108464.s002.tif]

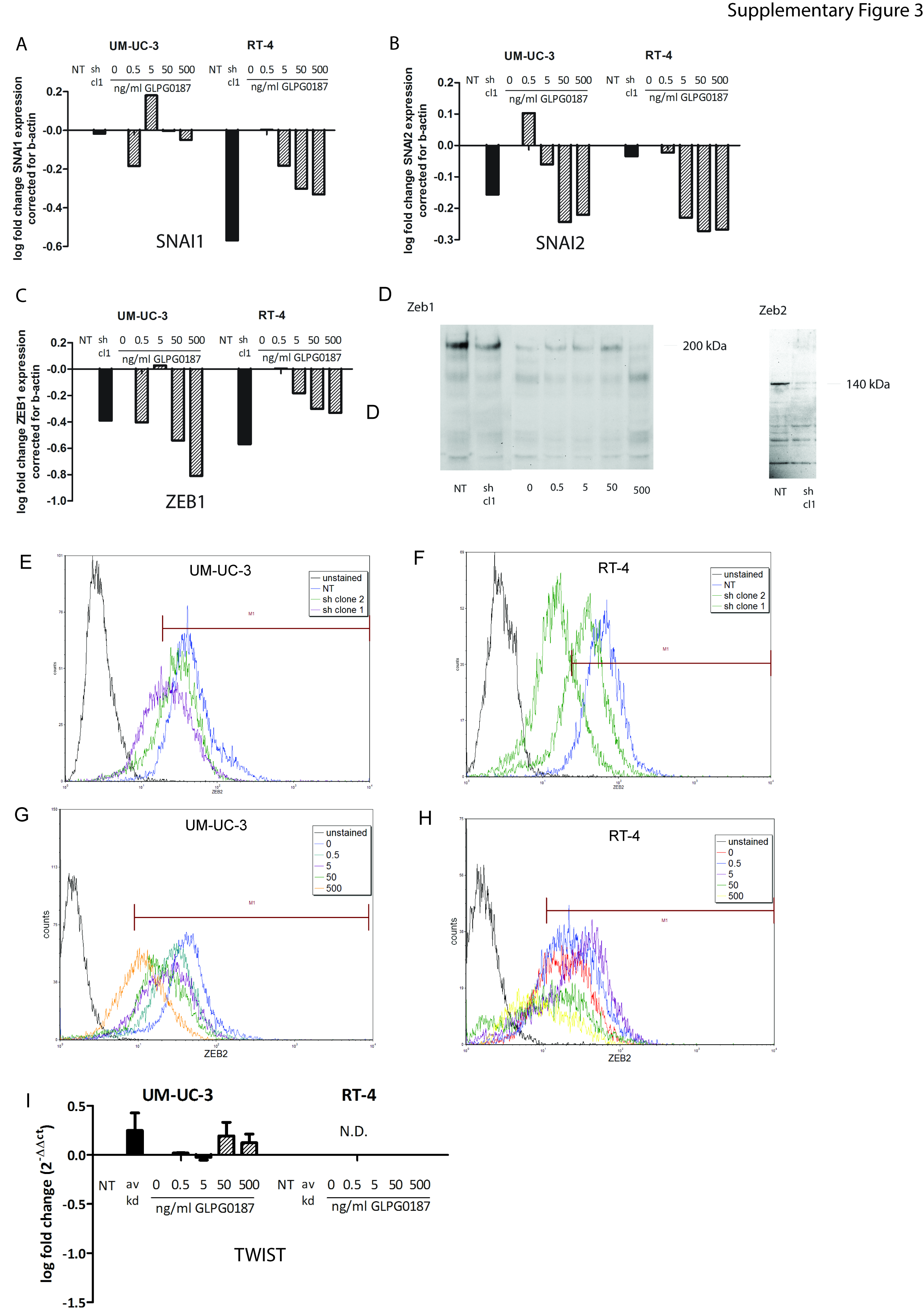

Supplement: Figure S3 — Protein levels of intracellular EMT markers. Densitometry analysis of the relative protein expression levels of SNAI1 (A), SNAI2 (B) and ZEB1 (C), measured with western blot analysis, compared to respectively NT or vehicle treated cells and corrected for b-actin expression levels in UM-UC-3 cells or RT-4 cells (respectively NT, sh clone 1, control and a concentration series of GLPG0187). Whole audiograms of ZEB1 and ZEB2 western blot analysis, displaying multiple additional bands (D). Representative images of cytometry plots of ZEB2 protein expression in UM-UC-3 NT and sh clones 1 and 2 (E) and ZEB2 protein expression in RT-4 NT and sh clones 1 and 2 (F). Representative images of cytometry plots of ZEB2 protein expression in UM-UC-3 cells (G) or RT-4 cells (H) treated with a dose-range of GLPG0187. Real time qPCR analysis of TWIST in UM-UC-3 and RT-4 cells (I). Relative expression levels are shown compared to respectively NT or non-treated cells. (TIF) [file pone.0108464.s003.tif]

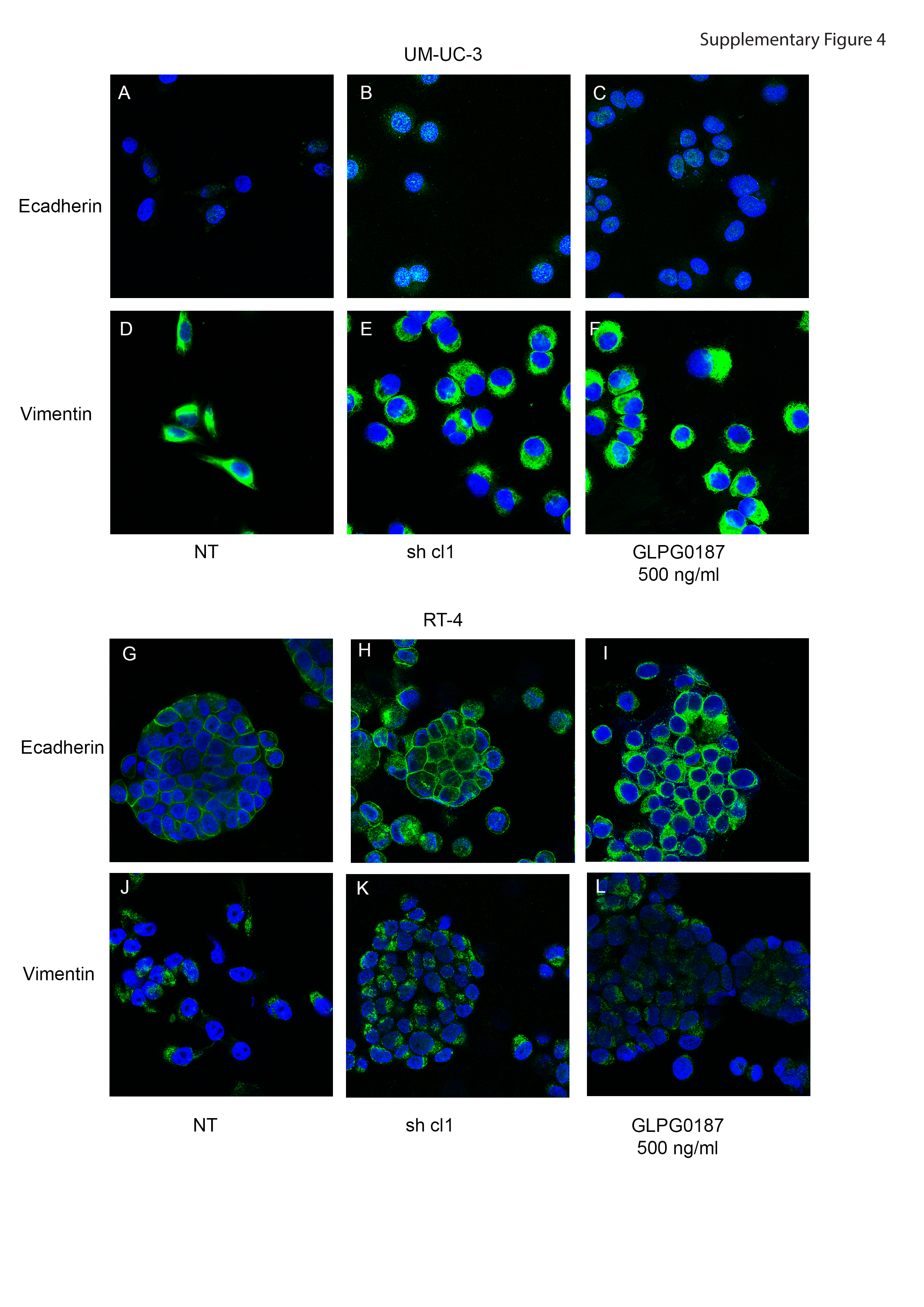

Supplement: Figure S4 — Immunofluorescence of E-cadherin and Vimentin. Representative confocal images of E-cadherin staining in UM-UC-3 NT (A), ITGAV knockdown clone 1 (B) and UM-UC-3 cells treated with 500 ng/ml GLPG0187 for 24 h (C) Representative confocal images of Vimentin staining in UM-UC-3 NT (D), ITGAV knockdown clone 1 (E) and UM-UC-3 cells treated with 500 ng/ml GLPG0187 for 24 h (F). Representative confocal images of E-cadherin staining in RT-4 NT (G), ITGAV knockdown clone 1 (H) and UM-UC-3 cells treated with 500 ng/ml GLPG0187 for 24 h (I) Representative confocal images of Vimentin staining in RT-4 NT (J), ITGAV knockdown clone 1 (K) and RT-4 cells treated with 500 ng/ml GLPG0187 for 24 h (L). (TIF) [file pone.0108464.s004.tif]

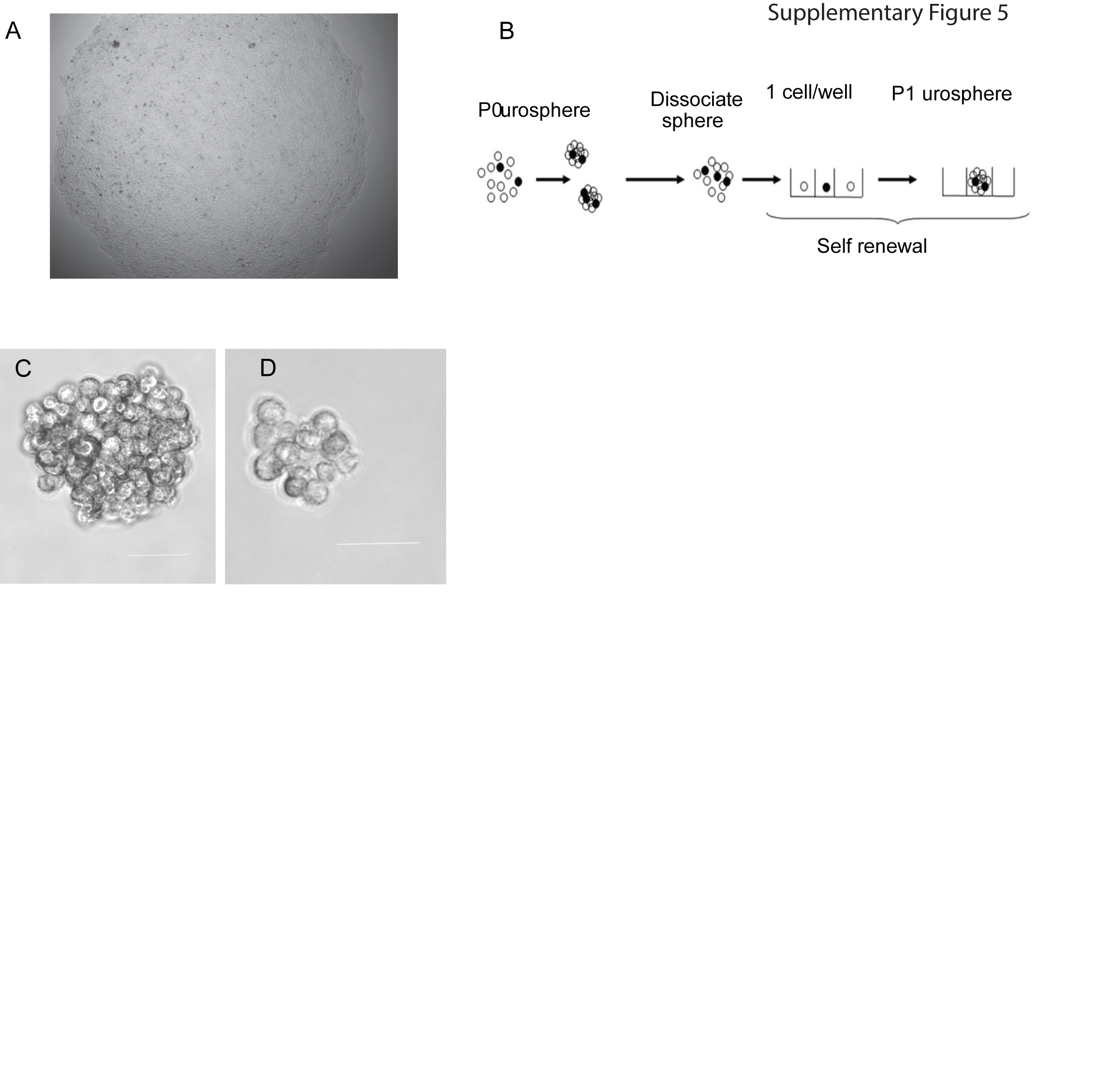

Supplement: Figure S5 — Tumor-initiating cell characteristics. Representative image of a colony in a clonogenic assay of UM-UC-3 cells 14 days after seeding (5x magnification) (A). Schematic representation of the urosphere protocol, adapted from Bisson et al [35]. (B) Representative images of UM-UC-3 NT (C) and ITGAV knockdown (D) P0 urospheres 10 days after seeding. Scale bar represents 50 µm (20x magnification). (TIF) [file pone.0108464.s005.tif]

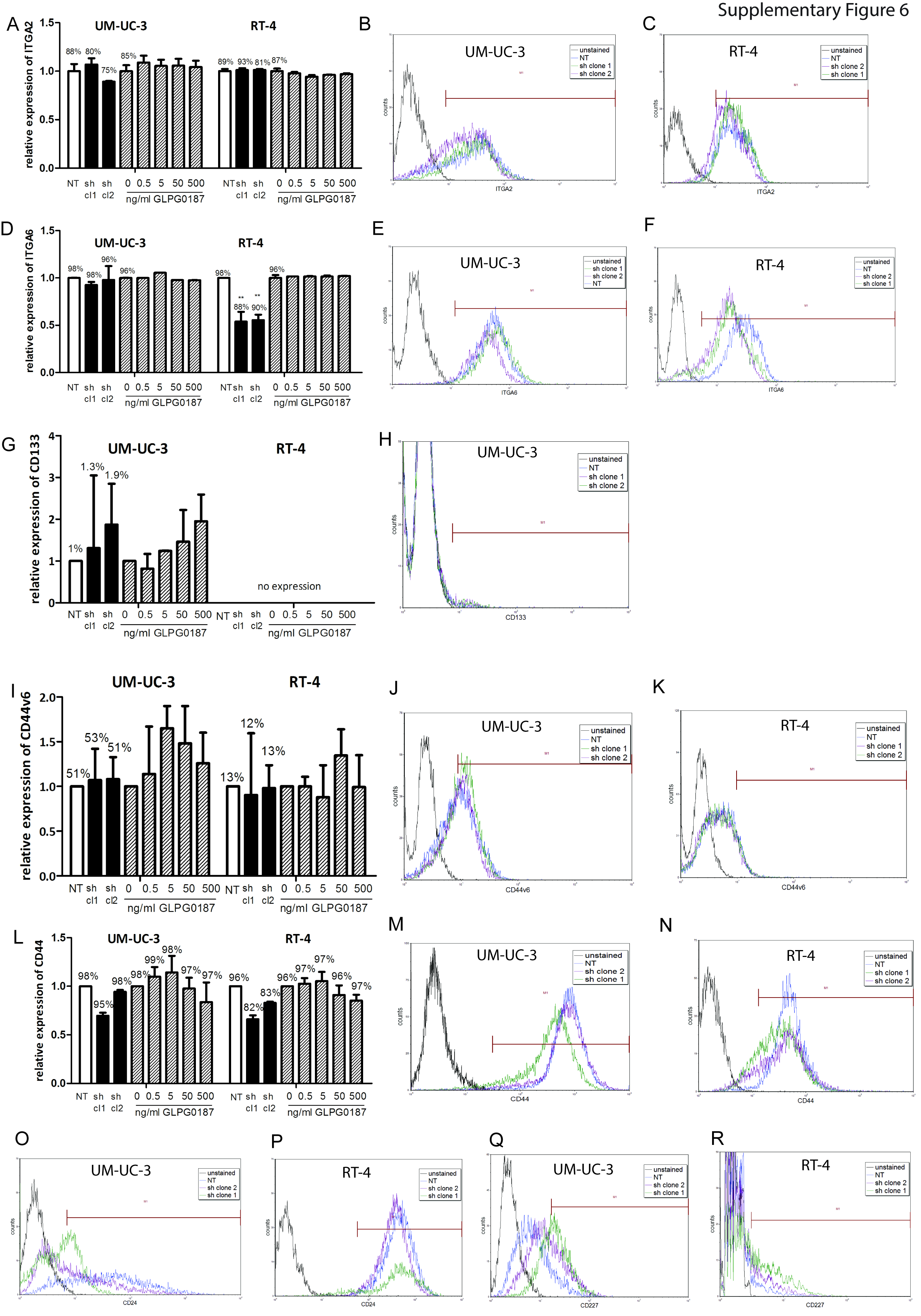

Supplement: Figure S6 — Expression levels of markers. Expression levels of ITGAV knockdown clones 1 and 2 were compared to control cells transduced with a non-targeting short hairpin (NT). Cells were treated with GLPG0187 concentration series for 48 h. Data were normalized to the NT or control conditions and are presented as mean ± SEM. Relative expression levels of ITGA2-FITC (percentage of positive cells * mean fluorescence intensity (A) with representative cytometry plot for UM-UC-3 (B) or RT-4 cells (C). Relative expression levels of ITGA6-APC (percentage of positive cells * mean fluorescence intensity (D) with representative cytometry plot for UM-UC-3 (E) or RT-4 cells (F). Relative expression levels of CD133-APC (percentage of positive cells * mean fluorescence intensity (G) with representative cytometry plot for UM-UC-3 (H). Relative expression levels of CD44v6-FITC (percentage of positive cells * mean fluorescence intensity (I) with representative cytometry plot for UM-UC-3 (J) or RT-4 cells (K). Relative expression levels of CD44v6-PE (percentage of positive cells * mean fluorescence intensity (L) with representative cytometry plot for UM-UC-3 (M) or RT-4 cells (N). Representative cytometry plot for CD24 expression in UM-UC-3 (O) or RT-4 cells (P). Representative cytometry plot for CD227 expression in UM-UC-3 (Q) or RT-4 cells (R). (TIF) [file pone.0108464.s006.tif]

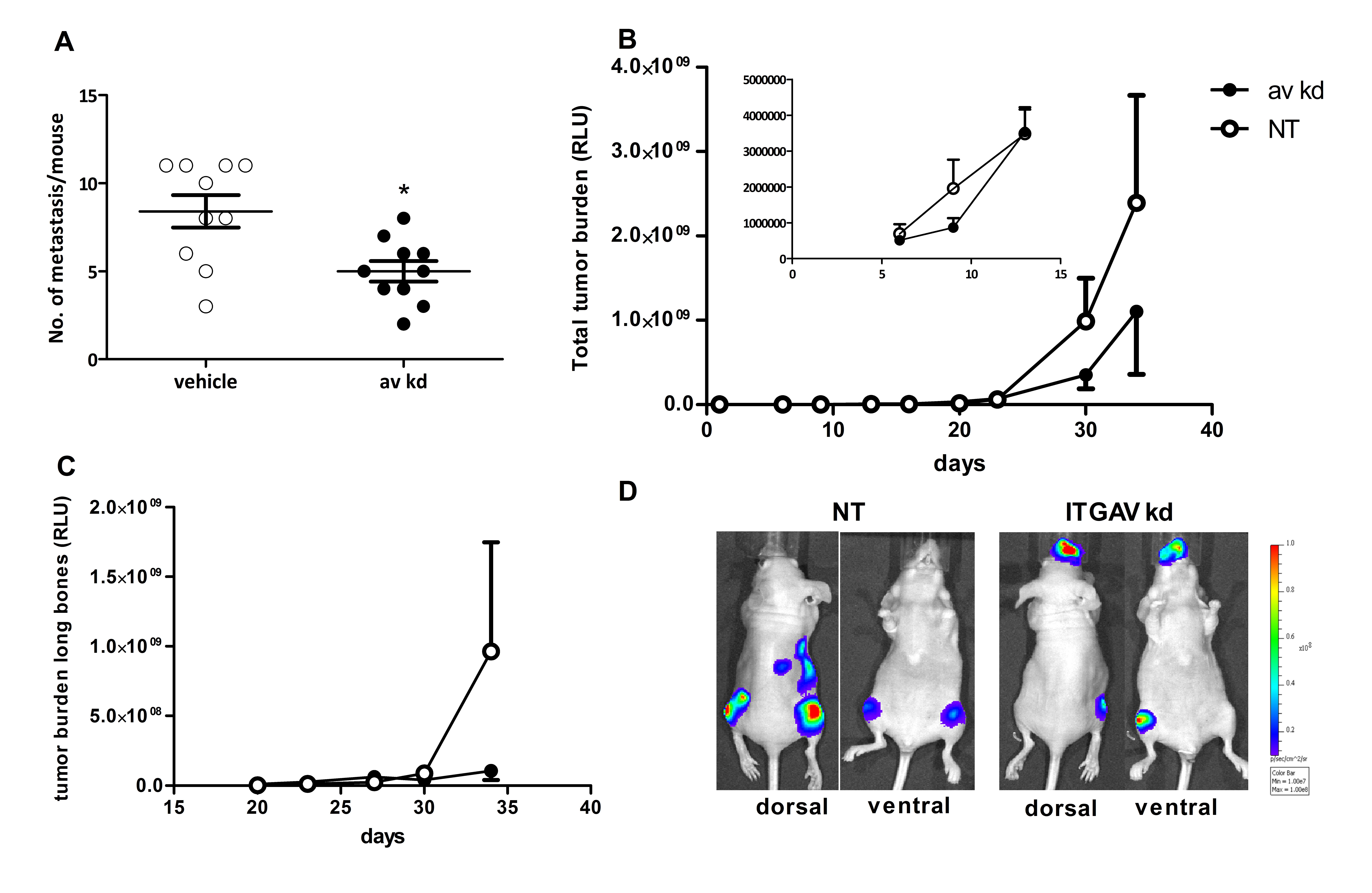

Supplement: Figure S7 — ITGAV knockdown in UM-UC-3luc2 cells affects metastatic growth in a preclinical model. A) Number of metastases/mouse after intra-cardiac inoculation of either αv-kd-UM-UC-3luc2 or NT-UM-UC-3luc2 cells. B) Total tumor burden and C) tumor burden in long bones after intra-cardiac inoculation of either αv-kd-UM-UC-3luc2 (closed circles) or NT-UM-UC-3luc2 cells (open circles) D) representative images of mice at day 34 after inoculation. (JPG) [file pone.0108464.s007.jpg]
