# Supplementary material for: Targeting of Alpha-V Integrins Reduces Malignancy of Bladder Carcinoma
Source: PLoS One. 2014 Sep 23;9(9):e108464. doi: 10.1371/journal.pone.0108464 (PMC4172769; doi:10.1371/journal.pone.0108464)
Supplement: Table S1 — Short hairpin RNAi constructs. UM-UC-3luc2 and RT-4 cell lines were transduced with short hairpin RNAi constructs against ITGAV or scrambled non-targeting (NT) shRNA derived from Sigma’s MISSION library. (DOC) [file pone.0108464.s008.doc]

| **Name** | **SIGMA Mission Library TRC no.** | **(target) sequence** |
| --- | --- | --- |
| Scrambled non-targeting (NT) | TRC1/1.5 | 5′-CCGGCAACAAGATGAAGAGCACCAACTCGAGTTGGTGCTCTTCATCTTGTTGTTTTT-3′ |
| ITGAV sh clone 1 | TRCN000010769 | CGACAGGCTCACATTCTACTT |
| ITGAV sh clone 2 | TRCN0000003240 | CTCTGTTGTATATCCTTCATT |

**Supplementary table 1 Short hairpin RNAi constructs**

UM-UC-3luc2 and RT-4 cell lines were transduced with short hairpin RNAi constructs against ITGAV or scrambled non-targeting (NT) shRNA derived from Sigma’s MISSION library.
