# Supplementary material for: Targeting of Alpha-V Integrins Reduces Malignancy of Bladder Carcinoma
Source: PLoS One. 2014 Sep 23;9(9):e108464. doi: 10.1371/journal.pone.0108464 (PMC4172769; doi:10.1371/journal.pone.0108464)
Supplement: Table S2 — Antibodies with application, supplier and location. (DOCX) [file pone.0108464.s009.docx]

| **protein** | **Antibody** | **Application** | **Supplier** | **Address** |
| --- | --- | --- | --- | --- |
| ITGAV | anti-human ITGAV (CD51)-PE | FACS | R&D Systems | Minneapolis, MN, USA |
| CDH1( E-cadherin) | FITC mouse anti-E-cadherin | FACS | BD Bioscience Pharmingen | Breda, The Netherlands |
| CDH2 (N-cadherin) | N-cadherin-PE | FACS | AbCaM | Cambridge, UK |
| CD133 | CD133/1-APC | FACS | Miltenyi Biotec | Leiden, The Netherlands |
| CD227 | CD227-APC | FACS | R&D Systems | Minneapolis, MN, USA |
| CD24 | PE anti-human CD24 | FACS | BD Biosciences Pharmingen | Breda, The Netherlands |
| ITGA2 | Anti-human CD49b-FITC | FACS | BD Biosciences Pharmingen | Breda, The Netherlands |
| ITGA6 | Monoclonal anti-human ITGA6-APC | FACS | R&D Systems | Minneapolis, MN, USA |
| CD44 | Anti-human CD44-APC | FACS | BD Biosciences Pharmingen | Breda, The Netherlands |
| CD44v6 | Human CD44v6 -APC | FACS | R&D Systems | Minneapolis, MN, USA |
| SNAI1 | Snail (C15D3) Rabbit mAb | Western Blot | Cell Signaling Technology | Boston, MA, USA |
| SNAI2 | Slug (C19G7) Rabbit mAb | Western Blot | Cell Signaling Technology | Boston, MA, USA |
| ZEB1 | TCF8/ZEB1 (D80D3) Rabbit mAb | Western Blot | Cell Signaling Technology | Boston, MA, USA |
| ZEB2 | Anti-Smad Interacting Protein 1 antibody - C-terminal | FACS intracellular | AbCaM | Cambridge, UK |
| Vimentin | Vimentin (D21H3) XP® Rabbit mAb | Immunofluorescence | Cell Signaling Technology | Boston, MA, USA |
| E-cadherin | E-Cadherin (24E10) Rabbit mAb | Immunofluorescence | Cell Signaling Technology | Boston, MA, USA |
| Rabbit IgG (H+L) | Alexa Fluor® 488 Goat Anti-Rabbit IgG (H+L) Antibody | Immunofluorescence and indirect FACS | Life Technologies | Bleiswijk, The Netherlands |

**Supplementary table 2** Antibodies with application, supplier and location.
