# Supplementary material for: Targeting of Alpha-V Integrins Reduces Malignancy of Bladder Carcinoma
Source: PLoS One. 2014 Sep 23;9(9):e108464. doi: 10.1371/journal.pone.0108464 (PMC4172769; doi:10.1371/journal.pone.0108464)
Supplement: Table S3 — Exon-spanning real-time PCR primers. Exon-spanning real-time PCR primers were designed with Primer Express software (Applied Biosystems, Rotkreuz, Switzerland). KRT20 expression was measured with Taqman primer/probe set Hs00300643_m1 from Life Technologies. (DOC) [file pone.0108464.s010.doc]

| **primer** | **sequence** |
| --- | --- |
| GAPDH forward | 5’ -GACAGTCAGCCGCATCTTC- 3’ |
| GAPDH reverse | 5’-GCAACAATATCCACTTTACCAGAG- 3’ |
| Snai1 forward | 5′-TGCAGGACTCTAATCCAAGTTTACCC-3′ |
| Snai1 reverse | 5′-GTGGGATGGCTGCCAGC-3′ |
| Snai2 forward | 5′-TGTGTGGACTACCGCTGC-3′ |
| Snai2 reverse | 5′-TCCGGAAAGAGGAGAGAGG-3′ |
| Twist forward | 5′-TGTCCGCGTCCCACTAGC-3′ |
| Twist reverse | 5′-TGTCCATTTTCTCCTTCTCTGGA-3′ |
| POU5F1 forward | 5’-AATACCTCAGCCTCCAGCAGATG- 3’ |
| POU5F1 reverse | 5’-TGCGTCACACCATTGCTATTCTTC- 3’ |
| Nanog forward | 5’- AATACCTCAGCCTCCAGCAGATG-3’ |
| Nanog reverse | 5’- TGCGTCACACCATTGCTATTCTTC-3’ |
| Zeb1 forward | 5’ – AGCAGTGAAAGAGAAGGGAATGC-3’ |
| Zeb1 reverse | 5’ – GGTCCTCTTCAGGTGCCTCAG-3’ |
| Zeb2 forward | 5’ – GACCTGGACGTGAAGGAAAA-3’ |
| Zeb2 reverse | 5’ – GGCACTTGCAGAAACACAGA-3’ |
| BMI-1 forward | 5’ – TGATGTGTGTGCTTTGTGGAG-3’ |
| BMI-1 reverse | 5’ – GGTCTGGTCTTGTGAACTTGG-3’ |
| Sox2 forward | 5’ – TACAGCATGTCCTACTCG -3’ |
| Sox2 reverse | 5’ – GAGGAAGAGGTAACCACA-3’ |

Supplementary Table 3 Exon-spanning real-time PCR primers designed with Primer Express software (Applied Biosystems, Rotkreuz, Switzerland).

KRT20 expression was measured with Taqman primer/probe set Hs00300643_m1 from Life Technologies.
