# Supplementary material for: Targeting of Alpha-V Integrins Reduces Malignancy of Bladder Carcinoma
Source: PLoS One. 2014 Sep 23;9(9):e108464. doi: 10.1371/journal.pone.0108464 (PMC4172769; doi:10.1371/journal.pone.0108464)
Supplement: Materials and Methods S1 — Materials and methods describing Annexin V/Propidium Iodide Apoptosis Assay, proliferation assay and Immunofluorescence staining. (DOC) [file pone.0108464.s011.doc]

Supplementary Materials and Methods

**Annexin V/Propidium Iodide Apoptosis Assay**

For apoptotic analysis, harvested cells were stained with Annexin V/propidium iodide (Alexa Fluor 488 Annexin V/Dead Cell Apoptosis Kit; Invitrogen), incubated for 15 minutes according to the manufacturer's protocol. Samples were analyzed with FACSCalibur2 (BD Biosciences) and FCS Express 3 software (DeNovo Software).

**Proliferation assay**

Cells were seeded in a 96-wells plate at a density of 1500 cells/well. Cells were allowed to grow for 24 h and then treated with vehicle or GLPG0187 in a concentration range of 0.5, 5, 50 and 500 ng/ml. Twenty microliters of MTS (Celltiter 96 Aqueous One Solution Cell proliferation assay, Promega Corporation, Madison, WI, USA) was added 24, 48 and 72 h after treatment and mitochondrial activity was measured at 490 nm after 2 h incubation at 37°C, using a Versamax microplate reader (Molecular devices).

**Immunofluorescence staining**

Cells were seeded on a glass chamber (NT and control cells), and allowed to grow for 1 day. Floating cells (ITGAV knock down or GLPG0187 treated cells) were placed on glass using cytospin. Cells were subsequently fixed with 4% paraformaldehyde for 5 min. After blocking for 1h in blocking buffer (0.2% normal goat serum), cells were stained with anti-E-cadherin (1:200) or anti-vimentin (1:100) for 1h. After washing with PBS, cells were stained with Alexa488 conjugated secondary antibody (goat-anti rabbit) 1:200 for 2h at RT in the dark. Vectashield containing DAPI was used for the mounting. Images were taken using a Confocal Laser Scanning Microscope (Leica TCS SP8).

**Intracardiac Inoculation:**

A single cell suspension of 1x10^5^ UM-UC-3luc2 cells/100 µl PBS was injected into the left cardiac ventricle of 4-week old nude mice as described previously [34]. n=10 per experimental group. Bioluminescence imaging (BLI)was performed using the IVIS Lumina Imaging System (Caliper LifeSciences, USA) [34]. Images were quantified with Living ImageTM and values expressed as relative light units (RLU). Tumor take was measured as the % of mice with BLI foci.
